# A multi-modal single-cell and spatial expression map of metastatic breast cancer biopsies across clinicopathological features

**DOI:** 10.1038/s41591-024-03215-z

**Published:** 2024-10-30

**Authors:** Johanna Klughammer, Daniel L. Abravanel, Åsa Segerstolpe, Timothy R. Blosser, Yury Goltsev, Yi Cui, Daniel R. Goodwin, Anubhav Sinha, Orr Ashenberg, Michal Slyper, Sébastien Vigneau, Judit Jané‐Valbuena, Shahar Alon, Chiara Caraccio, Judy Chen, Ofir Cohen, Nicole Cullen, Laura K. DelloStritto, Danielle Dionne, Janet Files, Allison Frangieh, Karla Helvie, Melissa E. Hughes, Stephanie Inga, Abhay Kanodia, Ana Lako, Colin MacKichan, Simon Mages, Noa Moriel, Evan Murray, Sara Napolitano, Kyleen Nguyen, Mor Nitzan, Rebecca Ortiz, Miraj Patel, Kathleen L. Pfaff, Caroline B. M. Porter, Asaf Rotem, Sarah Strauss, Robert Strasser, Aaron R. Thorner, Madison Turner, Isaac Wakiro, Julia Waldman, Jingyi Wu, Jorge Gómez Tejeda Zañudo, Diane Zhang, Nancy U. Lin, Sara M. Tolaney, Eric P. Winer, Edward S. Boyden, Fei Chen, Garry P. Nolan, Scott J. Rodig, Xiaowei Zhuang, Orit Rozenblatt-Rosen, Bruce E. Johnson, Aviv Regev, Nikhil Wagle

**Affiliations:** 1 Klarman Cell Observatory, Broad Institute of Harvard and MIT, Cambridge, MA USA; 2 Department of Medical Oncology, Dana-Farber Cancer Institute, Boston, MA USA; 3 Harvard Medical School, Boston, MA USA; 4 Department of Chemistry and Chemical Biology, Harvard University, Cambridge, MA USA; 5 Baxter Laboratory in Stem Cell Biology, Department of Microbiology and Immunology, Stanford University School of Medicine, Stanford, CA USA; 6 Department of Media Arts and Sciences, McGovern Institute, Massachusetts Institute of Technology, Cambridge, MA USA; 7 Center for Cancer Genomics, Dana-Farber Cancer Institute, Boston, MA USA; 8 Faculty of Engineering, Gonda Brain Research Center and Institute of Nanotechnology, Bar-Ilan University, Ramat Gan, Israel; 9 Broad Institute of Harvard and MIT, Cambridge, MA USA; 10 Center for Immuno-Oncology, Dana-Farber Cancer Institute, Boston, MA USA; 11 School of Computer Science and Engineering, The Hebrew University of Jerusalem, Jerusalem, Israel; 12 Racah Institute of Physics, The Hebrew University of Jerusalem, Jerusalem, Israel; 13 Faculty of Medicine, The Hebrew University of Jerusalem, Jerusalem, Israel; 14 Department of Media Arts and Sciences, Massachusetts Institute of Technology, Cambridge, MA USA; 15 Department of Biological Engineering, Massachusetts Institute of Technology, Cambridge, MA USA; 16 Department of Biology, Koch Institute for Integrative Cancer Research, Massachusetts Institute of Technology, Cambridge, MA USA; 17 Howard Hughes Medical Institute, Chevy Chase, MD USA; 18 Department of Brain and Cognitive Sciences, Massachusetts Institute of Technology, Cambridge, MA USA; 19 K. Lisa Yang Center for Bionics, Massachusetts Institute of Technology, Cambridge, MA USA; 20 Department of Stem Cell and Regenerative Biology, Harvard University, Cambridge, MA USA; 21 Department of Pathology, Brigham and Women’s Hospital, Boston, MA USA; 22 Department of Pathology, Dana-Farber Cancer Institute, Boston, MA USA; 23 Department of Physics, Harvard University, Cambridge, MA USA; 24 Present Address: Gene Center and Department of Biochemistry, Ludwig Maximilians Universität München, Munich, Germany; 25 Present Address: Department of Microbiology, Immunology and Genetics, Faculty of Health Sciences, Ben-Guiron University, Beersheba, Israel; 26 Present Address: AstraZeneca R&D, Boston, MA USA; 27 Present Address: Department of Microbiology, Immunology, and Cancer Biology, University of Virginia, Charlottesville, VA USA; 28 Present Address: Genentech, Inc., South San Francisco, CA USA

**Keywords:** Breast cancer, Cancer genomics, Data integration, Transcriptomics

## Abstract

Although metastatic disease is the leading cause of cancer-related deaths, its tumor microenvironment remains poorly characterized due to technical and biospecimen limitations. In this study, we assembled a multi-modal spatial and cellular map of 67 tumor biopsies from 60 patients with metastatic breast cancer across diverse clinicopathological features and nine anatomic sites with detailed clinical annotations. We combined single-cell or single-nucleus RNA sequencing for all biopsies with a panel of four spatial expression assays (Slide-seq, MERFISH, ExSeq and CODEX) and H&E staining of consecutive serial sections from up to 15 of these biopsies. We leveraged the coupled measurements to provide reference points for the utility and integration of different experimental techniques and used them to assess variability in cell type composition and expression as well as emerging spatial expression characteristics across clinicopathological and methodological diversity. Finally, we assessed spatial expression and co-localization features of macrophage populations, characterized three distinct spatial phenotypes of epithelial-to-mesenchymal transition and identified expression programs associated with local T cell infiltration versus exclusion, showcasing the potential of clinically relevant discovery in such maps.

## Abstract (web summary)

Single-nucleus and single-cell RNA sequencing plus spatial profiling with four methods of core biopsies from 60 patients with metastatic breast cancer reveal patient-specific gene expression programs of breast cancer metastases that are maintained across time, site of metastasis and spatial profiling method, with spatial phenotypes correlating with microenvironmental features.

## Main

Although malignant cells are the defining feature of cancers, tumors comprise malignant and non-malignant cells interacting in complex ecosystems that shape disease progression^1^. Understanding these interactions has potential for clinical translation. For example, although tumor-infiltrating lymphocytes (TILs) are generally associated with favorable prognosis, there is substantial heterogeneity^2^. In primary breast cancer (BC), TILs are predictive of response to neoadjuvant chemotherapy and improved survival in triple-negative breast cancer (TNBC) and human epidermal growth factor receptor 2-positive (HER2^+^) BC, but their impact in hormone receptor-positive (HR^+^) BC remains unclear and may depend on distinct states of malignant cells or TILs^3^.

Recent advances in single-cell and spatial profiling enable interrogation of tissue ecosystems at unprecedented resolution. However, few studies have focused on metastatic disease, likely due to sample limitations, including availability, size and diversity. Moreover, the panoply of available methods with distinct design parameters poses challenges for users in choosing methods^4,5^. As part of the Human Tumor Atlas Network (HTAN)^6^, we used single-cell and single-nucleus RNA sequencing (sc/snRNA-seq) and four distinct spatial expression methods (CODEX^7,8^, targeted ExSeq^9^, MERFISH^10–12^ and Slide-seq^13^) to profile tumor biopsies from a cohort of patients with metastatic breast cancer (MBC), the leading cause of cancer-related death among women worldwide^14^, toward informing practical application of these methods and refining understanding of MBC.

## Results

### Single-cell and spatial expression profiling of clinical variables

**Fig. 1:**
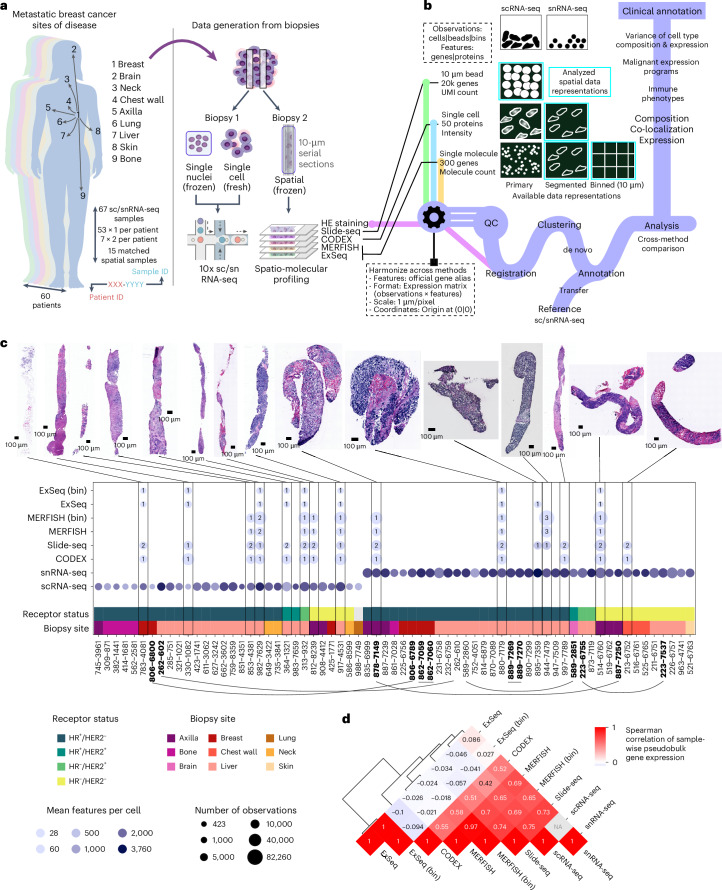
Profiling of MBC biopsies using scRNA-seq, snRNA-seq and four spatial expression methods. **a**, Schematic illustrating sample acquisition and data generation. Core biopsies dedicated to research were embedded in OCT or subjected to scRNA-seq. Per biopsy, one fresh or frozen core was used for scRNA-seq or snRNA-seq, respectively. For matching spatial profiling, a second, OCT-embedded core from the same biopsy procedure was cut in two sets of five 10-μm serial sections for processing with four spatial expression methods (Slide-seq, CODEX, MERFISH and ExSeq) and H&E staining. **b**, Schematic illustrating the properties of the different produced data types, the data processing framework and the performed analysis. **c**, Overview statistics of the produced scRNA-seq, snRNA-seq and spatial expression data as well as exemplary H&E images for the core biopsies used in spatial profiling. Biopsy site and receptor status for each of the profiled cores is indicated as well as the number of profiled observations (cells, beads or bins) and the number of detected features (RNA species or proteins). The number of replicates for each spatial expression method and biopsy is indicated in the respective blobs. HR, hormone receptor (ESR1 and PGR). Biopsies from the same patient are indicated with bold font and connected through lines. **d**, Clustered heatmap depicting the pair-wise Spearman correlation of methods based on sample-wise pseudobulk expression.

**Extended Data Fig. 1:**
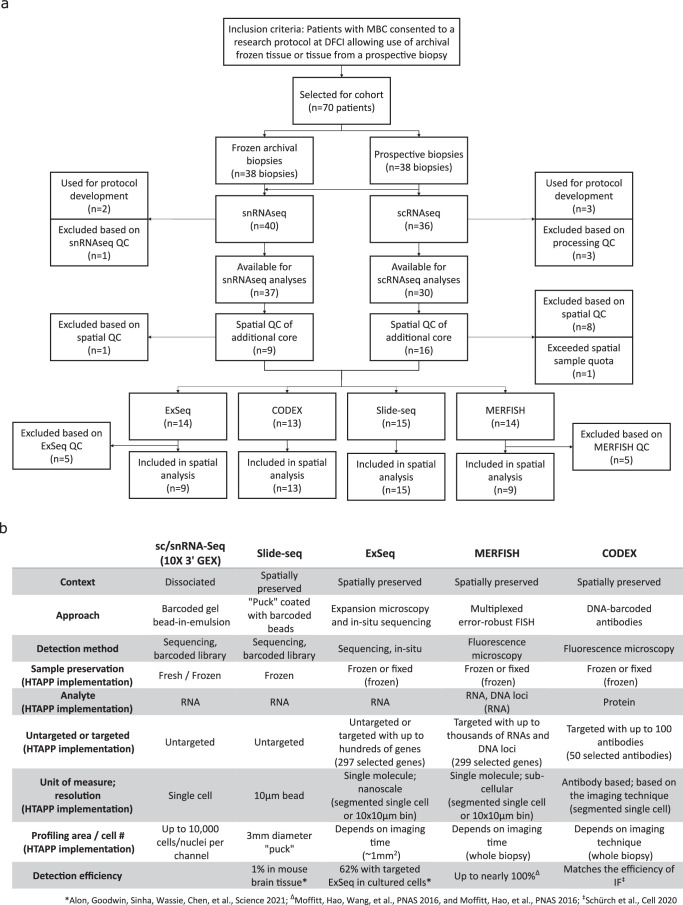
Overview of biopsy sample handling and profiling methods. **a**) Flow diagram outlining biopsy enrollment and allocation. **b**) Table outlining the key characteristics and design parameters of the profiling methods employed in this study.

To compare profiling methods and characterize cellular expression profiles of MBC biopsies, we created a comprehensive dataset covering relevant clinical variables and diverse profiling methods (Fig. 1a), along with an analysis framework to integrate the resulting data, by harmonizing features, data formats, positional resolution, coordinates and spatial registration (Fig. 1b and Methods), and we analyzed key features, including cell composition, gene expression programs, immune phenotypes and co-localization (Fig. 1b). We profiled 67 biopsies from 60 patients with MBC (30: scRNA-seq, 37: snRNA-seq) across receptor subtypes (44: HR^+^/HER2^−^, 3: HR^+^/HER2^+^, 3: HR^−^/HER2^+^, 16: HR^−^/HER2^−^) and frequent sites of disease (37: liver, 9: axilla, 7: breast, 5: bone, 3: chest wall, 3: neck, 1: brain, 1: lung, 1: skin; breast biopsies were collected from the primary site after MBC diagnosis) (Fig. 1a,c and Extended Data Fig. 1a). For 15 biopsies, we collected matching spatial data from serial sections of a second biopsy core from the same lesion/procedure, using up to four spatial methods and hematoxylin and eosin (H&E) staining (Fig. 1c, Extended Data Fig. 1a,b and Supplementary Tables 1 and 2).

The spatial techniques represent a range of design parameters (Extended Data Fig. 1b). Slide-seq profiles the whole transcriptome with near-cellular capture resolution using 10-μm beads (located independently of sample structure). CODEX, MERFISH and ExSeq target selected panels of proteins (CODEX) or RNAs (MERFISH and ExSeq) using imaging at single-cell, subcellular or super-resolution, respectively. Although ExSeq can be targeted or untargeted and MERFISH can potentially target up to thousands of RNAs, we designed a dedicated panel of 297 genes for MERFISH and ExSeq based on sc/snRNA-seq data and prior knowledge (Supplementary Table 3 and Methods).

We selected biopsies for tumor content and tissue quality and to cover a range of combinations of site and receptor status. We obtained high-quality Slide-seq and CODEX data from 15 of 15 and 13 of 13 biopsies, respectively, and MERFISH and ExSeq data from nine of 14 each (Fig. 1c and Extended Data Fig. 1b). The expert laboratories set sample quality control (QC) criteria individually (Methods). The comparatively low success rate of MERFISH is explained by its stringent inclusion criterion (Pearson’s *r* > 0.6 between MERFISH and matched sc/snRNA-seq pseudobulk profiles); for ExSeq, it was attributed to technical challenges (including tissue preservation, RNA quality and autofluorescence).

We analyzed single-molecule-resolution MERFISH and ExSeq data in two ways: aggregating signal per cell after cell segmentation or aggregating signal in 10 × 10-μm spatial bins. We analyzed Slide-seq by its native 10-μm beads and CODEX at the level of segmented cells (Fig. 1b,c). Analyzing single-molecule data by 10 × 10-μm bins generated coarser data in silico but avoided segmentation biases and allowed comparison to Slide-seq data while maintaining other method-specific properties (for example, detection sensitivity).

**Extended Data Fig. 2:**
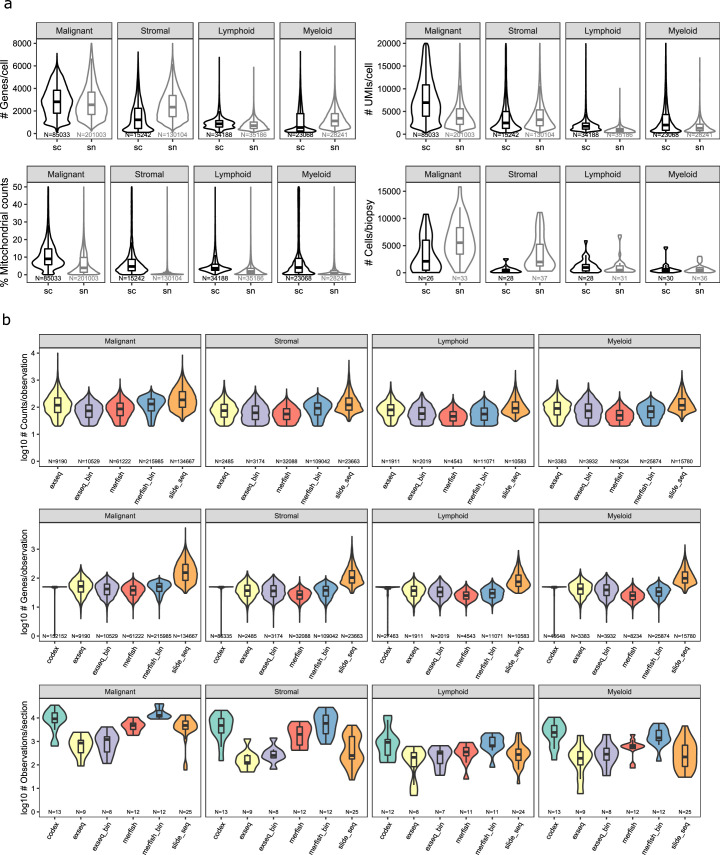
Quality statistics overview for sc/snRNA-Seq and spatial methods. **a**) Box- and violin plots depicting the distribution of the indicated quality measures for snRNA-Seq and scRNA-Seq data, stratified by cell type compartment (malignant, stromal, lymphoid, myeloid). N indicates cells or biopsy samples according to the axis labels. **b**) Box- and violin plots depicting the distribution of the indicated quality measures for the indicated spatial methods, stratified by cell type compartment (malignant, stromal, lymphoid, myeloid). N indicates observations (cells, beads, or bins) or tissue sections according to the axis labels.

As expected, the methods varied in the captured number of observations (cells/nuclei/beads/bins) and molecular features (genes/proteins) per observation (Fig. 1c, Extended Data Fig. 2a,b and Supplementary Tables 1 and 2). There was a higher number of observations and features per observation using snRNA-seq than scRNA-seq, whereas Slide-seq had a similar number of observations but many fewer features per observation. By definition, the number of features detected by approaches with predefined panels (MERFISH, ExSeq and CODEX) was lower per observation (Fig. 1c and Extended Data Fig. 2b). Between CODEX and MERFISH, which both captured the entire tissue section, CODEX yielded more observations per section than the segmented version of MERFISH but fewer than the binned version (Fig. 1c and Extended Data Fig. 2b). ExSeq, which captured only a small field of view (FOV) (<1 mm^2^), yielded the lowest number of observations per section in its segmented version, and this only slightly increased with binning (Fig. 1c and Extended Data Fig. 2b). Pseudobulk sample-wise expression profiles were correlated between all methods except ExSeq (Spearman *ρ* = 0.41 (CODEX versus scRNA-seq) to 0.75 (Slide-seq versus scRNA-seq), *ρ* = −0.1 to 0.086 (ExSeq)) (Fig. 1d). As expected, segmented and binned versions of MERFISH and ExSeq showed near-perfect correlations of 0.97 and 1, respectively (Fig. 1d).

### Clinical features are associated with cell type composition

**Fig. 2:**
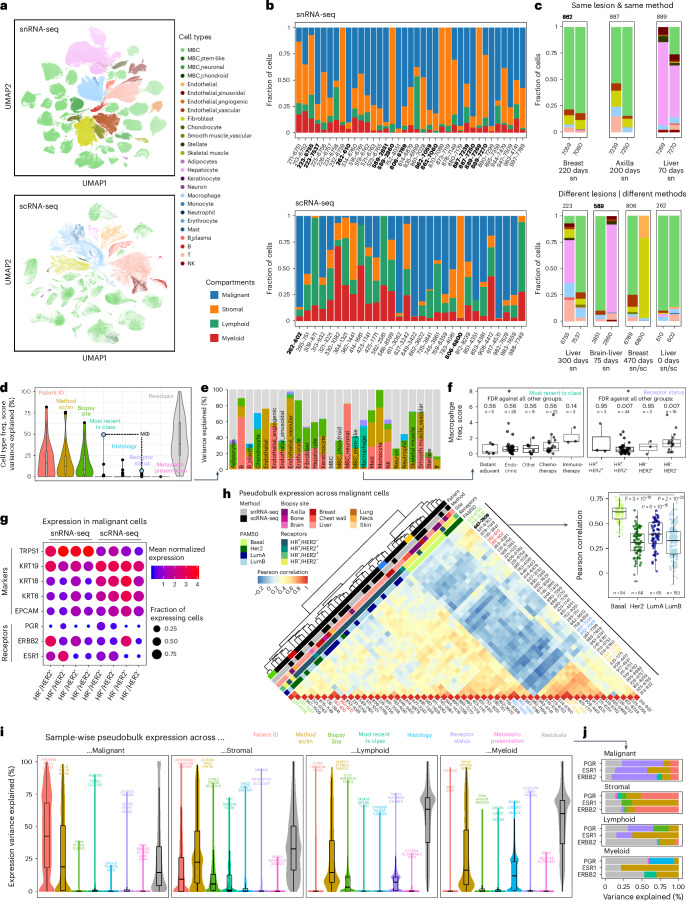
Cell type composition and expression variance in snRNA-seq and scRNA-seq data. **a**, UMAP representation of snRNA-seq and scRNA-seq data, colored by cell type. **b**, Stacked bar plots showing the cellular compartment composition for each sample in the snRNA-seq and scRNA-seq data. Samples that come from the same patient are highlighted in bold. **c**, Stacked bar plots showing the cell type composition for pairs of samples from the same patient. sc, scRNA-seq; sn, snRNA-seq. **d**, Violin and box plots representing the percent variance in cell type frequency explained by the indicated variable for each of the 26 annotated cell types (**e**). *n* = 26 cell types; tx, treatment. **e**, Stacked bar plots showing the percent variance in cell type frequency explained by the indicated variables for each of the 26 annotated cell types. **f**, Box plots with overlaid data points (=samples), representing the normalized macrophage frequency (Pearson’s contingency ratio) stratified by different properties of the two variables that explain variance in macrophage frequency (**e**). The significance of differences in ‘one against all others’ comparisons (two-sided Wilcoxon test, Benjamini–Hochberg correction) is indicated. *n* indicates the number of biopsy samples. **g**, Dot plots depicting the expression level (mean expression) and frequency (fraction of expressing cells) of malignant marker genes as well as disease-relevant BC biomarkers across malignant cells, grouped by =profiling method and receptor status. **h**, Clustered heatmap of pair-wise correlations between pairs of pseudobulk expression profiles representing each sample’s malignant cell population, corrected for profiling method using ComBat (Methods). Inset: box plots overlaid with individual data points (=sample combinations as in the heatmap) showing the pair-wise Pearson correlation across samples within PAM50 groups. The significance of differences between the basal and all other groups (two-sided Wilcoxon test) is indicated. **i**, Violin and box plots representing for all genes the percent variance in normalized expression levels across sample-wise and compartment-wise pseudobulk profiles, explained by the indicated variable. The top 3–5 genes are indicated. *n* = 26,539 genes. **j**, Stacked bar plots showing the percent variance in normalized expression levels across sample- and compartment-wise pseudobulk profiles, explained by the indicated variables from **i** for the three receptor status defining genes, *ESR1*, *PGR* and *ERBB2*.

**Extended Data Fig. 3:**
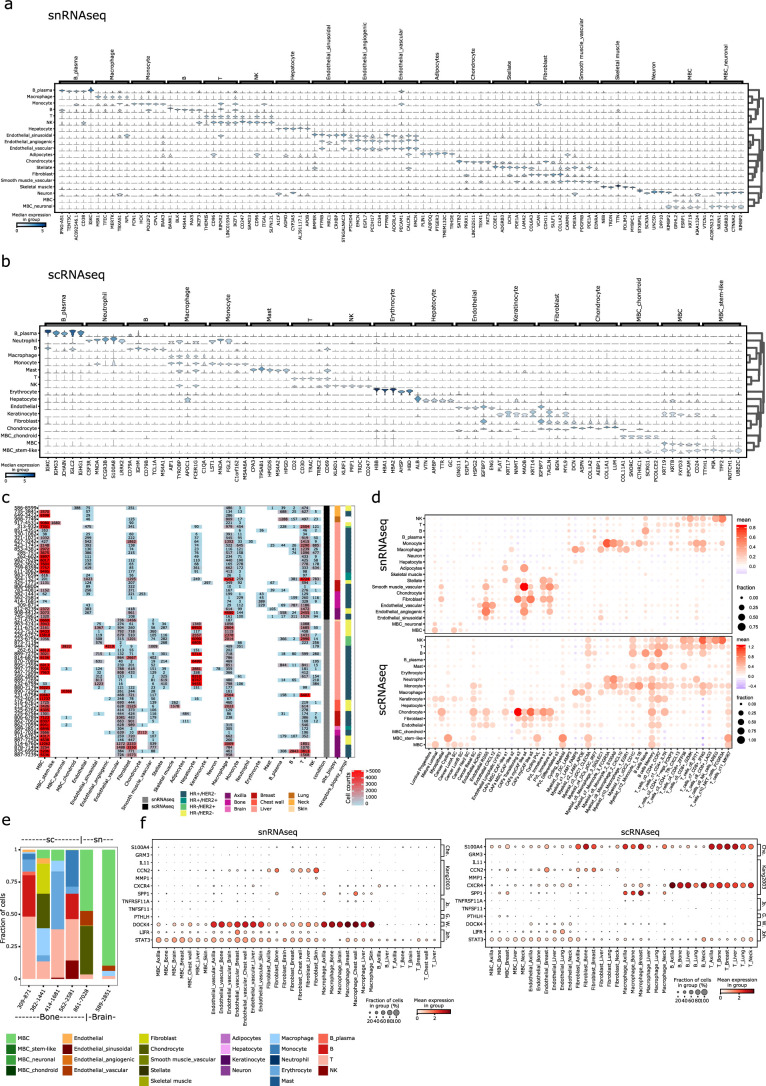
Cell type characterization of the sc/snRNA-Seq data. **a, b**) Stacked violin plots depicting the expression of the top 5 cell type marker genes for each of the indicated cell types, detected by 1 vs. all differential expression analysis for the snRNA-Seq data (panel **a**) and scRNA-Seq data (panel **b**). **c**) Heat map depicting the number of cells of each cell type detected in each of the samples. The color scale corresponds to the indicated respective number of cells. **d**) Dot plots depicting the expression level (mean expression) and frequency (fraction of expressing cells) of the indicated previously published cell subtype signatures^17^ across cells of the annotated broader cell types. **e**) Stacked barplot showing the cell type composition for biopsies of bone and brain metastasis. **f**) Dot plots depicting the expression of genes reported to be implicated in bone metastasis (Che.: Chen2017^27^, Kang2003^24^, Jo.:Jones2006^25^, G.:Guise1996^23^, W.:Westbrook2018^28^, Joh.:Johnson2016^26^) across cell types and metastatic sites covered in the snRNA-Seq (left) and scRNA-Seq (right) dataset, respectively.

We annotated cell types in sc/snRNA-seq using a semi-automated approach (Methods and Fig. 2a), combined with examination of the top five marker genes for each cell type (Extended Data Fig. 3a,b). Although most cell types were identified in snRNA-seq and scRNA-seq, some were detected only in snRNA-seq (adipocytes, neurons, some endothelial subsets, stellate cells and smooth and skeletal muscle cells) or scRNA-seq (neutrophils, mast cells, erythrocytes and keratinocytes) (Fig. 2a and Extended Data Fig. 3a,c), largely consistent with previous reports^15,16^. Several cell subtype signatures from scRNA-seq of primary BC^17^ scored highly in the expected cell types (Extended Data Fig. 3d). As expected, most of the scRNA-seq-derived signatures scored higher in scRNA-seq than in snRNA-seq.

Although most malignant cells displayed epithelial-like expression profiles, in a few samples we observed chondroid (sample 586-8599), stem-like (sample 917-4531) or neuronal (samples 944-7479 and 890-7299) expression profiles (Extended Data Fig. 3a–c). Interestingly, these were associated with unique clinicopathologic characteristics. The sample with stem-like expression profiles came from the patient with the cohort’s shortest overall survival from initial diagnosis (<2 years), despite presenting with stage I disease and receiving appropriate treatment. The sample with a chondroid expression profile was the only biopsy with metaplastic histology, and the clinical pathology independently described chondroid differentiation. Metaplastic BC is a rare and heterogenous subtype associated with poor prognosis overall^18^ and poor response to cytotoxic chemotherapy^19,20^ but in which preliminary data suggest the possibility of responsiveness to immunotherapy with frequent PD-L1 expression^21^ and a subset of patients with exceptional responses to combined checkpoint blockade on a phase 2 trial^22^. Although anecdotal, these vignettes demonstrate that expression features recovered by sc/snRNA-seq can be consistent with rare clinicopathologic features and may warrant further investigation.

Biopsy composition by four major compartments (malignant, stromal, myeloid and lymphoid) varied across samples but, overall, scRNA-seq captured a higher fraction of immune cells, and snRNA-seq had greater representation of malignant and stromal cells (Fig. 2b), which are prone to death during dissociation^15^. To investigate sources of composition differences, we analyzed the biopsies from seven patients with two biopsies each. In one, two cores from the same procedure were profiled with snRNA-seq and scRNA-seq. These showed the expected bias toward enriched immune cells in scRNA-seq and malignant and stromal cells in snRNA-seq (Fig. 2c). In three patients, the paired biopsies were obtained from the same lesion at different timepoints (70–220 days apart), and each pair showed relatively similar compositions overall but with changes in T cell and macrophage frequencies (two decrease, one increase). In contrast, in each of the three patients in whom the paired biopsies were from different lesions or sites, we observed more substantial differences, largely driven by hepatocytes and fibroblasts. Irrespective of method, biological factors, such as individual, time, lesion and site, can have substantial effects on composition.

We examined the impact of scRNA-seq (four biopsies) versus snRNA-seq (one biopsy) in bone biopsies, a clinically relevant metastatic site that yields lower content biopsies (Extended Data Fig. 3e,f). Although scRNA-seq captured malignant cells in only two of four, snRNA-seq captured the malignant compartment well but yielded fewer immune cells (Extended Data Fig. 3e), suggesting that snRNA-seq might be more suitable when prioritizing malignant cell profiling, and scRNA-seq might be more suitable when prioritizing associated immune cells. Notably, expression of genes previously reported to be implicated in bone metastasis^23–28^ was detected across all biopsy sites (not bone specific) and was rather cell type specific (Extended Data Fig. 3f), with two exceptions (*SPP1* and *CCN2*), which were more highly expressed in axilla, bone and breast macrophages and fibroblasts, respectively (Extended Data Fig. 3f). We also examined the ability of snRNA-seq to profile brain metastases, a clinically relevant site underrepresented in genomic datasets. snRNA-seq captured both malignant cells and tumor microenvironment well, anecdotally supporting this approach (Extended Data Fig. 3e).

Next, we systematically quantified the contributions of biological, clinical and technical variables to variability in cell type composition (Methods). Patient ID, profiling method and site explained the most variability overall (Fig. 2d), but other variables had considerable effects on variation in particular cell types (Fig. 2e). Approximately 20% of the variability in chondrocytes was explained by histology, whereas variability in macrophages was explained by treatment class (~50%) and receptor status (~10%) (Fig. 2e). Higher macrophage abundance was associated with recent immunotherapy and with HR^−^/HER2^−^ disease (Fig. 2f).

### Clinical features explain variation in expression profiles

**Extended Data Fig. 4:**
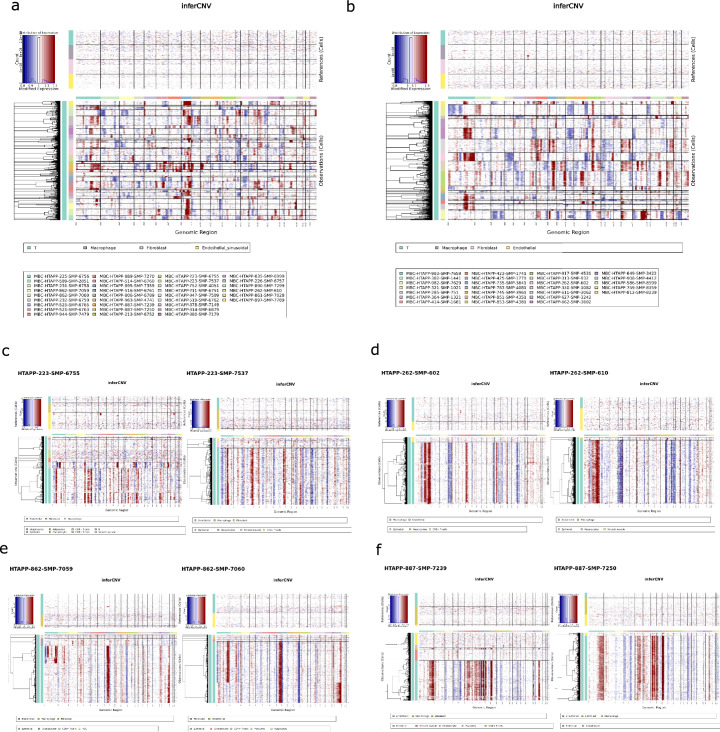
Copy number aberration (CNA) detected in the sc/snRNA-Seq data. **a,b**) CNA heatmaps across malignant cells, grouped by sample, for snRNA-Seq data (panel **a**) and scRNA-Seqdata (panel **b**) **c**) CNA heatmaps for both samples from patient 223. Samples were taken from different liver lesions, 300 days apart and processed with snRNA-Seq. **d**) CNA heatmaps for both samples from patient 262. Samples were taken from the same liver lesion at the same time but processed with snRNA-Seq and scRNA-Seq respectively. **e**) CNA heatmaps for both samples from patient 862. Samples were taken from the same breast lesion but 220 days apart, and processed with snRNA-Seq. **f**) CNA heatmaps for both samples from patient 887. Samples were taken from the same axilla lesion but 200 days apart, and processed with snRNA-Seq.

Although non-malignant cells clearly grouped by cell type across biopsies, malignant cells grouped first by patient (Fig. 2a) as previously described in scRNA-seq of solid tumors^17,29^, consistent with diverse patterns of inferred copy number aberrations (CNAs) between patients (Extended Data Fig. 4a,b). Conversely, biopsies from the same patient had congruent inferred CNAs across lesions (Extended Data Fig. 4c), profiling method (Extended Data Fig. 4d) and time (Extended Data Fig. 4e,f). Two biopsies taken 220 days apart (patient 862), with intervening therapy, retained the same subclonal structure, albeit with varying proportions (Extended Data Fig. 4e).

As expected, inter-patient variability in the expression of *ESR1*, *PGR* and *ERBB2* aligned well with clinical receptor status. Nevertheless, among estrogen receptor–positive (ER^+^) samples, *ESR1* expression was captured more robustly in snRNA-seq (Fig. 2g). Inter-patient variability in established epithelial BC marker genes (*EPCAM*, *KRT8*, *KRT18*, *KRT19* and *TRPS1*) was minimally impacted by receptor status but notably by profiling method (Fig. 2g).

**Extended Data Fig. 5:**
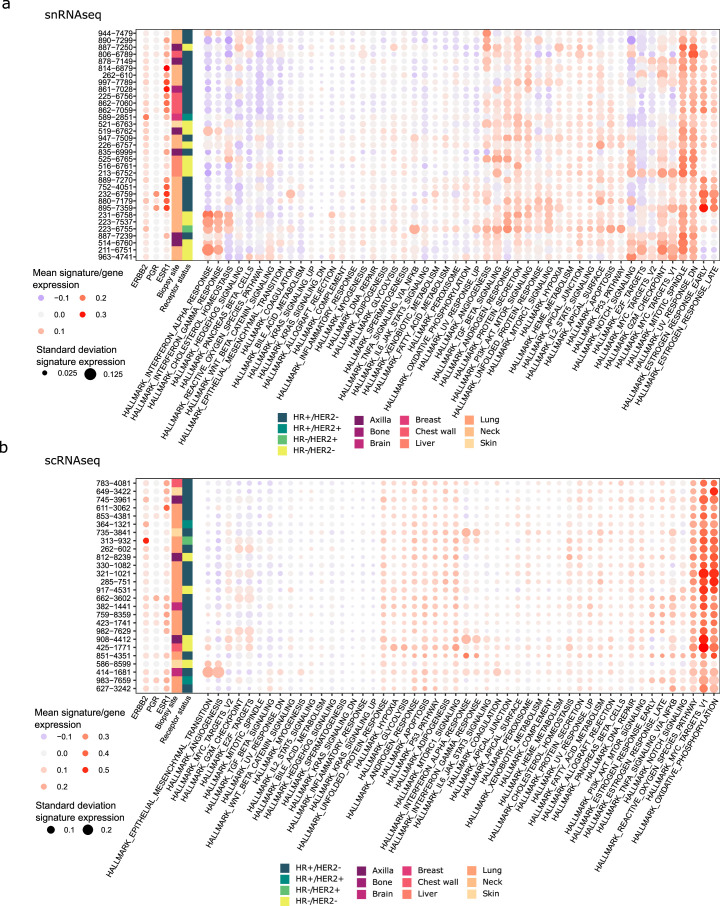
Expression of malignant hallmark signatures in the malignant sc/snRNA-Seq data. **a, b**) Dot plots depicting the expression level (mean expression) and variability (standard deviation) of the indicated hallmark gene sets in MSigDB^65,66^ across the malignant cells in each of the indicated samples, separately for snRNA-Seq (panel **a**) and scRNA-Seq data (panel **b**).

**Extended Data Fig. 6:**
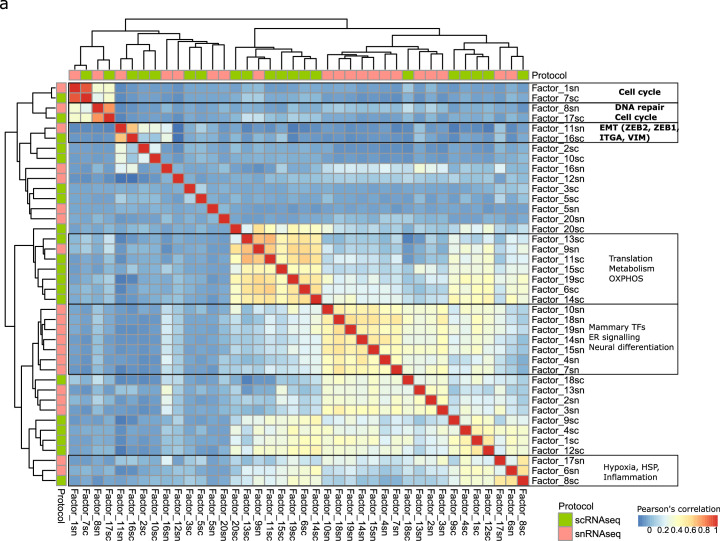
Malignant expression programs as identified by iNMF. **a**) Clustered heatmap of pairwise correlations across all 20 malignant expression programs, represented by relative gene importance, detected by iNMF in the snRNA-Seq data (frozen) and scRNA-Seq data (fresh), each.

At the level of expression programs, clustering malignant profiles by mean gene set enrichment analysis (GSEA) hallmark signature scores in malignant cells yielded clear grouping in snRNA-seq (for example, interferon response, estrogen response and MYC/G2M checkpoint groups) but less so in scRNA-seq, with few exceptions (for example, 414 and 586 scoring highly for epithelial-to-mesenchymal transition (EMT) and angiogenesis, respectively) (Extended Data Fig. 5). Clustering of 40 cross-sample malignant expression programs learned with integrative non-negative matrix factorization (iNMF)^30^ separately from snRNA-seq and scRNA-seq (Methods) revealed six clusters, five of which included programs derived from both methods. Three of these had highly correlated programs and congruent biological processes: two associated with cell cycle and the third with EMT (Extended Data Fig. 6). To further compare malignant cell states, we clustered pseudobulk profiles generated from the malignant cells of each biopsy. This revealed two major clusters: one predominantly comprised HR^+^ and LumA/B tumors and was enriched in liver biopsies (*P* = 0.0185, two-sided Fisher’s exact test), and the other predominantly comprised HR^−^/HER2^−^ biopsies, which further separated into basal-like and HER2-like subsets and was enriched in axilla biopsies (*P* = 4.92 × 10^−4^, two-sided Fisher’s exact test) (Fig. 2h). Basal-like biopsies formed a highly correlated exclusive subcluster (Fig. 2h), suggesting higher expression stability of the basal subtype, consistent with previous reports^31–33^. Notably, biopsies from the same patient grouped together, even in two cases where they changed from HR^+^ or HER2^+^ to HR^−^/HER2^−^, confirming the relative stability and patient specificity of malignant cell-intrinsic expression profiles through MBC disease progression, possibly due to the strong effect of CNAs on expression^34,35^.

**Extended Data Fig. 7:**
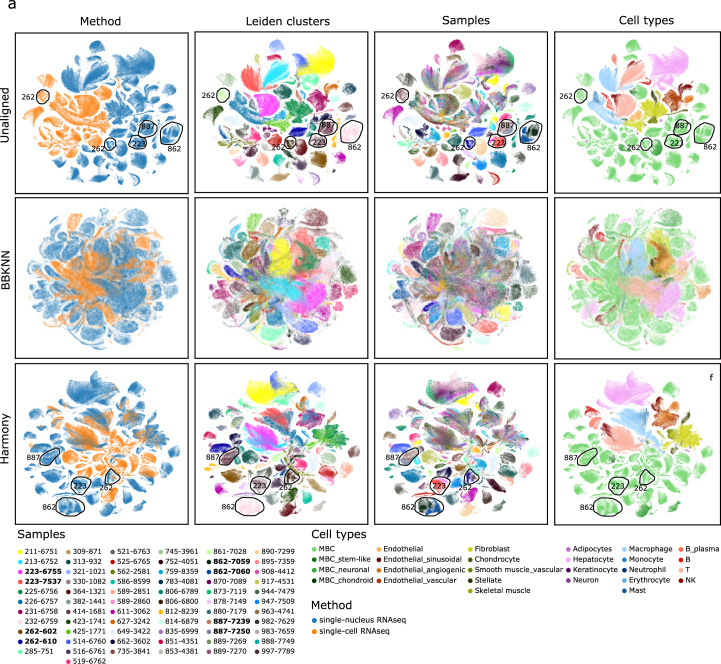
Integration of snRNA-Seq and scRNA-Seq data in low-dimensional space. **a**) UMAPs depicting all observations from the sc/snRNA-Seq data based on their unaligned, BBKNN integrated, or Harmony integrated, dimensionality-reduced transcriptomes. Colored by method, Leiden clusters (resolution: 0.4), samples, and cell types. Samples from the same patient are marked.

To dissect inter-patient expression variance in each compartment, we estimated, for each gene, the variability explained by clinical/technical covariates (Methods and Fig. 2i). These variables explained a large fraction of the inter-patient variance in intrinsic expression in the stromal (median, ~65%) and malignant (median, ~85%) compartments but much less in the immune compartments (median, ~30%). Consistent with our other observations, patient ID explained the most variance in the malignant compartment but played a negligible role in the immune compartments. Conversely, histology explained approximately 10% variance in the myeloid compartment but was negligible for all others. Across all compartments, profiling method explained a median of approximately 20–25% variance, consistent with previous reports^15,16^ (Fig. 2i and Extended Data Fig. 5). ComBat^36^ adequately corrected such ‘platform effects’ at the pseudobulk level, revealing relevant biology across methods (Fig. 2h), and Harmony^37^ (but not BBKNN^38^) produced an aligned embedding at the single-cell level that appropriately grouped non-malignant cells across patients/methods while maintaining biological variability in the malignant compartment (Extended Data Fig. 7).

Although receptor status explained a sizeable fraction of the expression variation of *PGR* (~56%), *ESR1* (~44%) and *ERBB2* (~68%) in the malignant compartment (Fig. 2j), it only explained substantial variance (>44%) in 34 other genes (Supplementary Table 4), some of which were reassuringly associated with one of the receptors. These included *STARD3*, *GRB7*, *MIEN1* and *LASP1*, which are adjacent to *ERBB2* on 17q12 and subject to co-amplification, and *MTA2*, whose expression is associated with ERα expression^39^. Others, including *TMSB4X* and *BECN1*, were previously associated with metastatic progression but not with BC receptor expression^40–42^, suggesting the potential to uncover novel associations.

These results show strong inter-patient variability of malignant expression profiles, with patient-specific profiles maintained during MBC progression through time, site and even changes in receptor status. In contrast, the expression profiles in the immune compartments showed only low levels of explainable variance by these characteristics. Additionally, although profiling methods have non-negligible effects on all compartments, these can be mostly addressed by data integration methods before comparing cell or gene profiles.

### Comparison of spatial expression profiling methods

**Extended Data Fig. 8:**
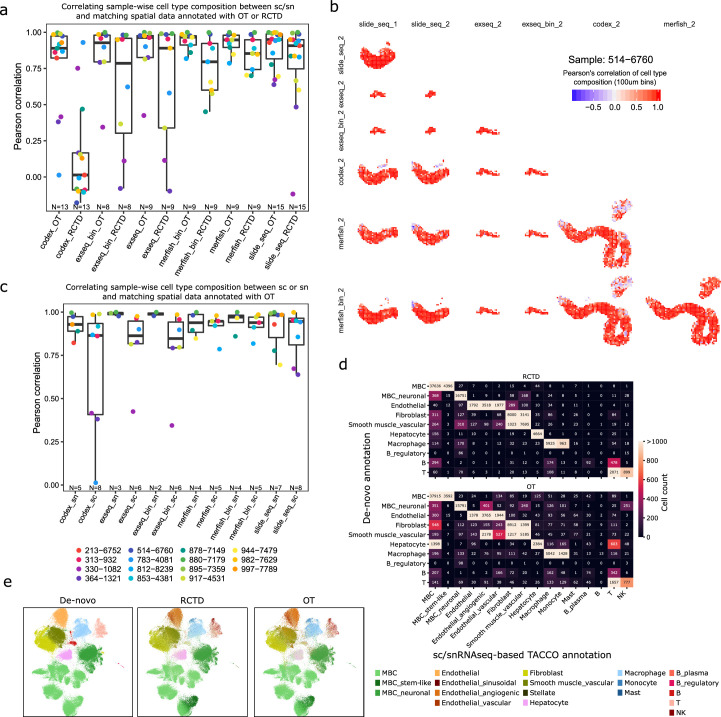
Correspondence of cell type composition across profiling methods and annotations. **a**) Boxplots depicting the correlation of cell type composition between sc/snRNA-Seq and spatial methods, for each biopsy, stratified by annotation method (TACCO-OT or RCTD). The individual data points are overlaid. N indicates number of sample-pairs. **b**) Spatial scatter plots displaying the correlation between cell type compositions within 100×100 μm bins as measured by the indicated pairs of methods in the 514-6760 biopsy. **c**) Boxplots depicting the correlation of cell type composition between sc/snRNA-Seq and spatial methods, for each biopsy, stratified by single-cell profiling method (snRNA-Seq or sc RNAseq). The individual data points are overlaid. **d**) Heatmap depicting for the segmented MERFISH data, the congruence of cell type annotations based on manual cluster analysis/marker expression (de-novo) and automated sn/scRNA-Seq-based annotation by TACCO-OT or RCTD, respectively. Numbers indicate the number of cells with the respective annotation combination. **e**) UMAPs of all cell-segmented MERFISH data based on their expression profiles, with observations colored by cell type as annotated based on cluster analysis/marker expression (de-novo), or annotation transfer from sc/snRNA-Seq using TACCO-OT or RCTD respectively.

Our experimental design enabled profiling serial sections of the same biopsy with up to four different methods (Fig. 1a). We used a common observation × features format for analysis, where observations corresponded to segmented cells (MERFISH, ExSeq and CODEX), beads (Slide-seq) or 10 × 10-μm bins (MERFISH (bin) and ExSeq (bin)), and features corresponded to RNA or protein sets denoted as the official gene alias for all methods (Fig. 1b and Methods). We scaled to a 1-μm-per-pixel positional resolution (Methods), registered to a common coordinate system, and applied quality filtering in a method-specific manner (Fig. 1b and Methods). We annotated cell types by label transfer from the matching sc/snRNA-seq using RCTD^43^ and TACCO-OT^44^ (Methods). TACCO-OT was selected for downstream analyses as it was better able to handle both count and non-count data (Extended Data Fig. 8a and Supplementary Figs. 1–5a,b).

**Fig. 3:**
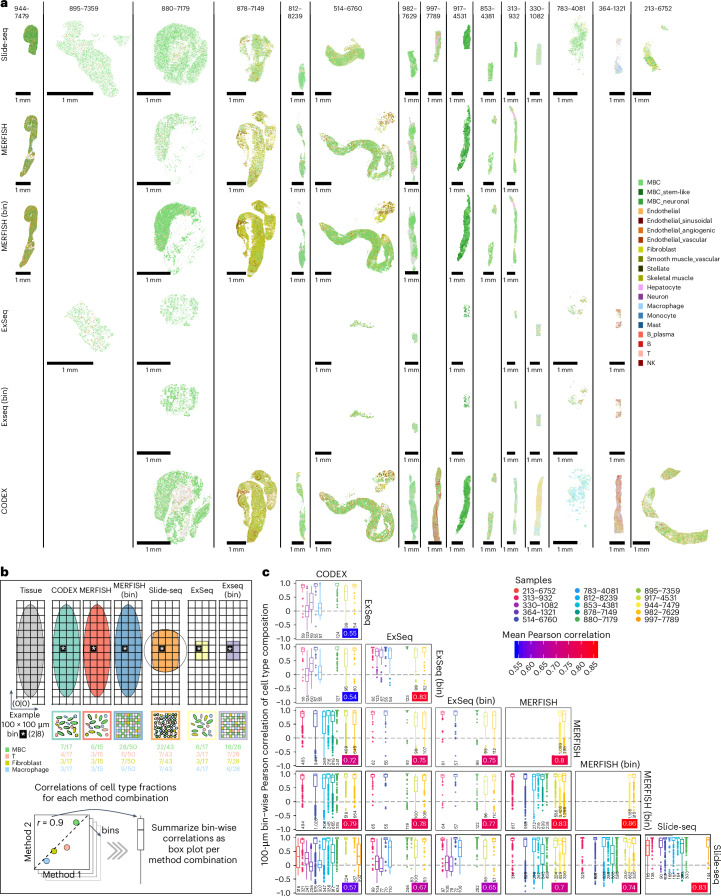
Spatial expression profiling of MBC biopsies. **a**, Overview of all spatial expression datasets covering all samples and methods included in this study. For each successful sample–method combination, a spatial scatter plot is shown where each observation (cell, bead and bin) is displayed and colored by its OT annotated cell type. Data for the same biopsy are spatially aligned and depicted at the same scale. A more detailed view of individual samples for which data are available from all spatial profiling methods is provided in Supplementary Figs. 1–5. **b**, Schematic illustrating the comparison by Pearson correlation of high-resolution cell type composition within spatially corresponding 100 × 100-μm bins across methods, within biopsies. An example for one bin (white star) within one biopsy is shown. **c**, Box plots displaying the correlations between cell type compositions within spatially corresponding 100 × 100-μm bins as measured by the indicated pairs of methods, displayed individually per biopsy. Correlations within the same method were calculated when technical replicates were available. The mean Pearson correlation for each pair of methods is indicated by the color-scaled inset. *n* indicates the number of 100 × 100-μm bins.

Spatial cell type maps appeared broadly congruent across serial sections profiled by different methods (Fig. 3a and Supplementary Figs. 1–5) but ranged in their FOV from the whole biopsy (MERFISH and CODEX) to a circular area with an approximately 3-mm diameter (Slide-seq) to approximately 1 mm^2^ (ExSeq). Binned MERFISH and ExSeq patterns matched the segmented ones but were more pronounced and less sparse, likely due to a combination of signal included in binning but lost due to non-assignment in segmentation as well as signal filling of cell-proximal extracellular space in binning. To assess the agreement between methods in local cell type organization, we calculated pair-wise correlations between methods based on cell type composition in aligned 100 × 100-μm bins (Fig. 3b,c and Extended Data Fig. 8b). Correlations were high across method combinations and samples (median Pearson’s *r* ≈ 0.9), except for three samples (330, 364 and 783) with no correlation (median, *r* ≈ 0) among any of the three methods (CODEX, ExSeq and Slide-seq) (Fig. 3c). These three samples did not pass MERFISH QCs, suggesting that more stringent pass/fail QC may be appropriate for other methods. Notably, cell type composition from spatial data also correlated well with sc/snRNA-seq across all methods (Pearson’s *r* ≈ 0.9) and slightly more highly with snRNA-seq than scRNA-seq (Extended Data Fig. 8c). This weakly supports snRNA-seq’s capacity to more faithfully represent cell type composition.

**Fig. 4:**
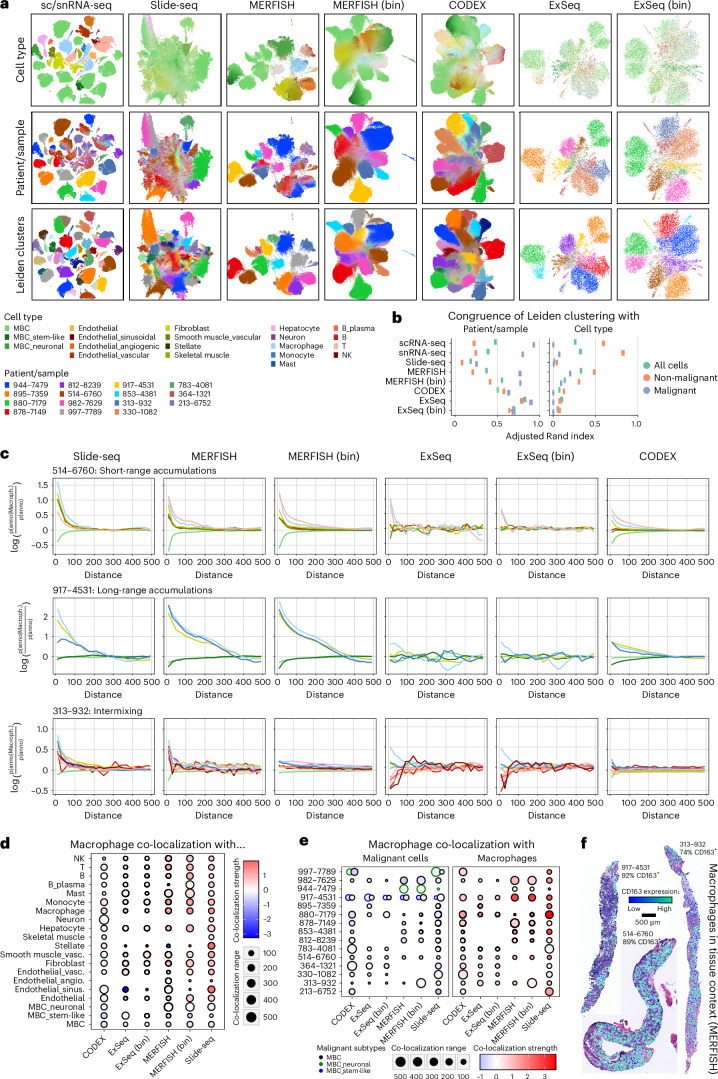
Recovering spatial and molecular signals across spatial expression profiling methods. **a**, UMAPs of all data across biopsies based on their expression profiles, generated with the indicated methods, with observations colored by TACCO-OT annotated cell type, patient/sample and Leiden clusters (resolution, 0.8). **b**, Error bar plot with mean ± s.d. showing the ARI quantifying cluster cohesion between Leiden clusters and patient/sample or cell type annotation across 10 bootstrapping iterations for each indicated method, as in **a**. ARI ranges between −1 and 1, where 1 indicates perfect agreement, 0 indicates a random agreement and −1 indicates completely different groupings. *n* = 10 bootstrapping iterations. **c**, Line plots depicting co-localization strength (*y* axis) of macrophages with all other measured cell types in dependence of distance (*x* axis), derived from the indicated data types in the indicated three biopsies, selected to represent three spatial co-localization phenotypes (short-range accumulation, long-range accumulations and intermixing). The distance is measured in μm. **d**, Dot plot displaying aggregated (mean across samples) co-localization range (size) and strength (color) of macrophages with all other cell types per method. Co-localization strength values lower than 0 indicate exclusion/repulsion. **e**, Dot plot displaying co-localization range (size) and strength (color) of macrophages with other macrophages or malignant cells for all samples and methods. Co-localization strength values lower than 0 indicate exclusion/repulsion. **f**, Spatial scatter plot of macrophages overlaid onto H&E images showing the expression levels of CD163 in the depicted macrophages, for the three example biopsies representing the three co-occurrence cases as in **c**, based on cell-segmented MERFISH data.

To assess each method’s cell or bin/bead-level profiles across samples, for each method (separately), we clustered all profiles, created a low-dimensional embedding for visualization and quantified the association of clusters with patient or cell type using the adjusted Rand index (ARI) (Fig. 4a,b and Supplementary Figs. 1d, 2d, 3d, 4d and 5d). sc/snRNA-seq and cell-segmented MERFISH grouped primarily by cell type and patient for normal and malignant cells, respectively (Fig. 4a,b). Conversely, binned or bead-based methods, where profiles are a composite across cells, reflected mostly a malignant cell, patient-specific signal, with less separation between clusters, and lower cell-type-driven separation of non-malignant cells, suggesting a dominating signal from prevalent malignant cells. CODEX clusters were also indistinct and mostly driven by patient, not cell type, possibly related to the antibody panel.

To assess each method’s capacity to capture local organization, we quantified, for each method, the co-localization of each cell type (as an ‘anchor cell’) versus all other cell types within 50 μm, showing consistency across methods (Supplementary Figs. 1c, 2c, 3c, 4c and 5c). To assess a broader distance range of 0–500 μm and systematically compare methods, we focused on macrophages, as they are present in most samples and are captured well by all methods. In general, Slide-seq, MERFISH and CODEX all captured short-range and long-range accumulations and intermixing of macrophages and other cell types similarly (Fig. 4c–e). ExSeq was often the weakest at capturing accumulation patterns (Fig. 4c). Notably, across all biopsies, macrophages preferentially co-localized with other macrophages and weakly avoided malignant cells (Fig. 4e). Visual inspection of macrophage distributions relative to the matching H&E images showed a distinct long-range pattern with macrophage islands and more homogenous short-range and intermixing phenotypes (Fig. 4e).

Overall, there was relatively high congruence among methods, but MERFISH showed several benefits: a large profiling area, clear spatial patterns and clear, sc/snRNA-seq-like clustering of cell profiles. As our MERFISH experiments only measure the expression of ~300 genes, we further assessed its ability to detect cell subsets without matching sc/snRNA-seq data. We compared clustering-based cell annotations obtained from segmented MERFISH to those from RCTD and TACCO-OT (Extended Data Fig. 8d,e). Although most were in agreement, MERFISH-based assignments lacked some granularity (only one endothelial cell label, joint T/NK labels) but captured other distinctions missing in sc/snRNA-seq, including a small cluster of B regulatory cells jointly expressing *FOXP3* and *FCRL5* (Extended Data Fig. 8e).

### Spatial profiling of tumor-associated macrophages

Tumor-associated macrophages (TAMs) are implicated in multiple stages of tumor progression and have prognostic implications in solid tumors, including BC^45–47^. However, their role, diversity and therapeutic potential remain only partially understood^48,49^. For example, although CD68^+^ leukocyte density alone was not found to be a prognostic biomarker in primary treatment-naive BC, a CD68^Hi^, CD4^Hi^, CD8^Lo^ immunoprofile was associated with reduced overall survival and recurrence-free survival^50^, and the presence of TAMs expressing the CD163 scavenger receptor was associated with adverse prognostic features in BC^51^. In our data, macrophages were ubiquitous across samples and measurement methods; their variable frequency across samples in our sc/sn composition analysis was highly explained by the most recent treatment class (with immunotherapy being weakly associated with higher macrophage frequencies) (Fig. 2d–f), and their spatial organization varied between samples and measurement methods when chosen as the ‘anchor cell’ (Fig. 4c–f and Supplementary Figs. 1c, 2c, 3c, 4c and 5c).

**Extended Data Fig. 9:**
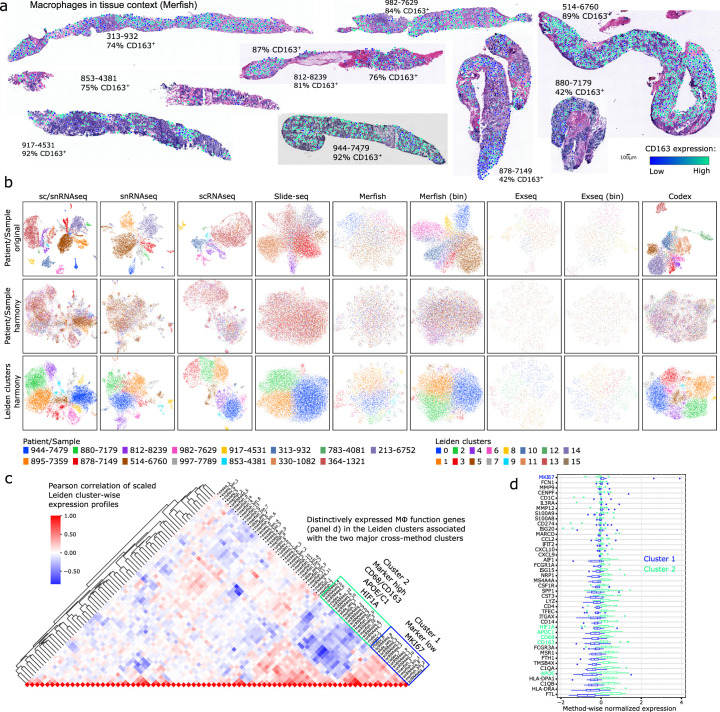
Characterization of macrophage subsets based on expression states across methods. **a**) Spatial scatter plot overlaid onto H&E images depicting the expression levels of CD163 in macrophages for all biopsies, based on cell-segmented MERFISH data. **b**) UMAPs of all observations confidently annotated as macrophages across biopsies based on their unaligned or harmony sample-integrated expression profiles, colored by sample/patient or Leiden clusters (resolution: 0.6). **c**) Clustered heatmap depicting the pairwise Pearson correlation of scaled Leiden cluster-wise expression profiles across methods. Method and cluster ID (as in panel b) are indicated. **d**) Boxplots depicting the expression of macrophage marker and function genes in the method-specific macrophage clusters grouped in cross-method cluster 1 (*N* = 15) and 2 (*N* = 20) according to panel c). Genes are ordered by the median difference between cross-method cluster 1 and 2. *N* = method-specific clusters.

Macrophage co-localization phenotypes (Fig. 4c,e) were neither specifically enriched nor depleted with expression of *CD163*, a key macrophage marker, with the three representative samples showing predominantly CD163^+^ macrophages (Fig. 4f). Moreover, most (73–93%) macrophages in the other biopsies profiled by MERFISH were also CD163^+^, with few intermixing CD163^−^ macrophages (Fig. 4f and Extended Data Fig. 9a). In the two notable exceptions (878 and 880), most macrophages were CD163^−^ (Extended Data Fig. 9a). Due to methodological limitations, these observations were only possible with MERFISH.

**Fig. 5:**
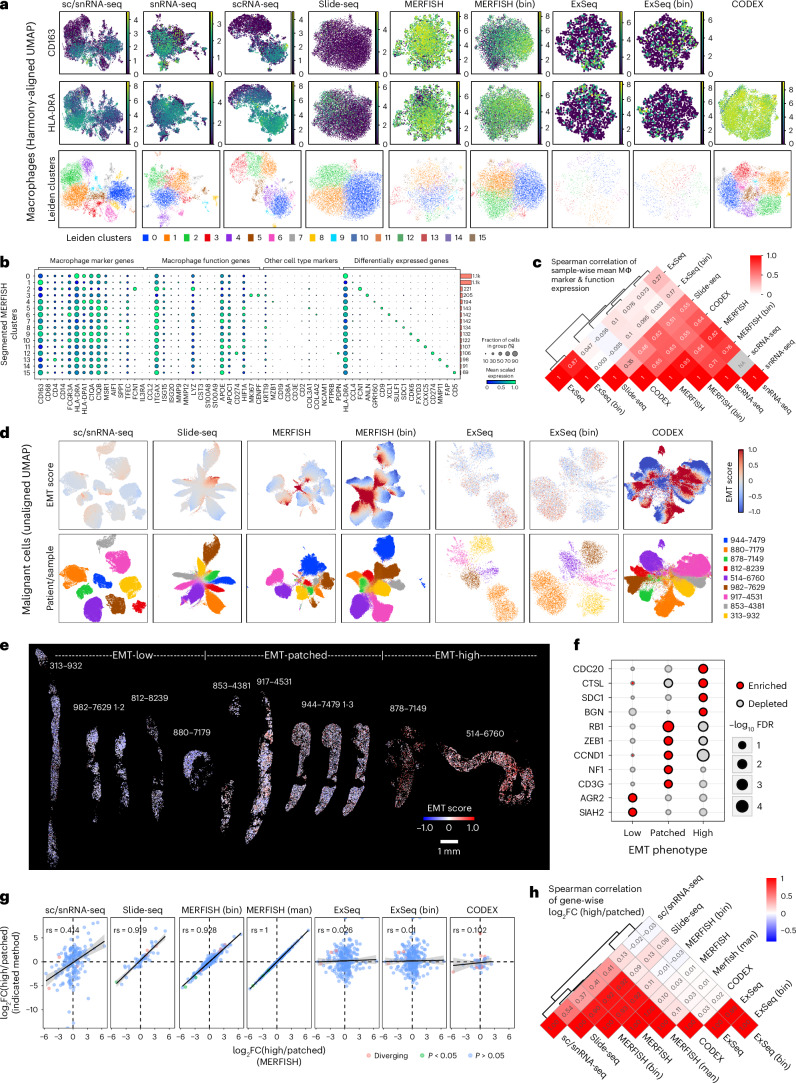
Characterizing macrophage and malignant expression phenotypes across spatial expression profiling methods. **a**, UMAPs of all observations confidently annotated as macrophages across biopsies based on their expression profiles, colored by log-normalized expression of CD163, log-normalized expression of HLA-DRA or Leiden clusters. **b**, Dot plot depicting the scaled expression (by gene, across clusters) and fraction of expressing cells of macrophage marker and function genes as well as marker genes for other cell types and differentially expressed genes between clusters as in **a** for cell-segmented MERFISH data. Side bar plots indicate the number of cells in each cluster. **c**, Clustered heatmap depicting the pair-wise Spearman correlation of methods based on sample-wise pseudobulk expression of macrophage marker and function genes as in **b**. **d**, UMAPs of all observations annotated as malignant cells across biopsies based on their expression profiles, colored by their EMT score expression (capped at −1 and 1 for comparability) or patient/sample. **e**, Spatial scatter plots of the cell-segmented MERFISH data where each cell is colored by its EMT score expression (capped at −1 and 1 for comparability). Samples are grouped into three spatial EMT phenotypes—EMT-high, EMT-low and EMT-patched—based on the distribution of the EMT signal across space. **f**, Dot plot depicting the differential expression significance (two-sided Welch’s *t*-test, Benjamini–Hochberg correction) of genes overexpressed in one of the three spatial EMT phenotypes (EMT-high, EMT-low and EMT-patched), as detected in the cell-segmented MERFISH data (**e**). **g**, Scatter plot relating the log fold changes of gene expression between EMT-high and EMT-patched samples as detected in cell-segmented MERFISH to the corresponding expression changes detected in the other indicated methods. The significance of differential expression was calculated by a two-sided Welch’s *t*-test and Benjamini–Hochberg correction. The Spearman correlation is indicated. Error bands indicate standard error. **h**, Clustered heatmap depicting the pair-wise Spearman correlation of methods based on gene-wise log fold changes between EMT-high and EMT-patched samples, defined as in **e** and related to **g**. FC, fold change; man, manual.

**Extended Data Fig. 10:**
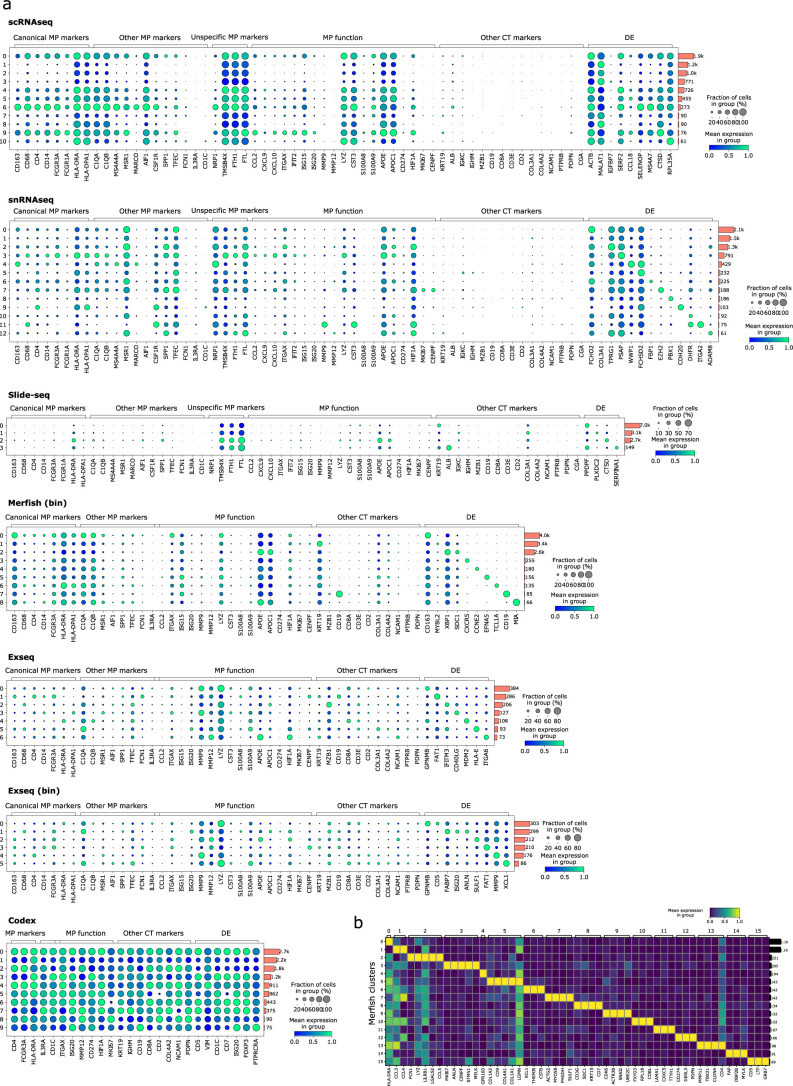
Macrophage subset marker gene expression. **a**) Dot plots depicting the scaled expression (by gene, across clusters) and fraction of expressing cells of macrophage marker and function genes as well as marker genes for other cell types and differentially expressed genes between clusters as in Extended Data Fig. 9b for all methods as indicated. Side-barplots indicate the number of cells in each cluster. **b**) Heatmap of scaled (across clusters) gene expression showing the top up to five differentially expressed genes (FDR < 0.05, log-fold change > 1.5) between the indicated cell-segmented MERFISH clusters.

To investigate broader macrophage expression states, we integrated all observations identified as macrophages using Harmony^37^ (within each method separately) and clustered them (Fig. 5a and Extended Data Fig. 9b). Using the same clustering resolution for all methods, we retrieved 4–15 clusters per method (Fig. 5a). Across all methods, there were two major clusters of highly correlated method-specific clusters: a CD163^+^ cluster with high expression of macrophage markers as well as *HIF1A* and *APOE*/*APOC1* and a CD163^−^ cluster associated with lower macrophage marker expression and expression of *MKI67* (Extended Data Fig. 9c,d). ExSeq and Slide-seq had much lower signal for macrophage markers overall (Fig. 5a and Extended Data Fig. 10a), but Slide-seq still showed moderate correlation to other methods. MERFISH was the most correlated with sc/snRNA-seq (*ρ* = 0.64–0.84; Fig. 5c) and demonstrated a similar pattern, with two large clusters along a single continuum (one CD163^+^, the other CD163^−^; Fig. 5a) as well as 13 small clusters of approximately 100 cells each, expressing shared macrophage markers and distinct cluster-defining genes associated with different states or functions, such as *ANLN* or *CDK6* (proliferation), *MMP11* (tissue remodeling) or *FCN1* (angiogenesis)^52^ (Fig. 5b and Extended Data Fig. 10b). Previous studies of primary BC described APOE-expressing macrophages as lipid-associated macrophages (LAMs), comprising up to 30–40% of all myeloid cells^17^. In our MERFISH data, the fraction of APOE-expressing macrophages varied from 24% to 85% of all macrophages (mean, 48%).

### Spatial interaction and expression phenotypes

We examined the spatial organization of malignant cells considering their expression of the EMT program initially identified with scRNA-seq (Extended Data Fig. 6a). We observed intra-patient and inter-patient variability in EMT signals among the malignant cells across all methods (Fig. 5d). Although cells from samples with low and high EMT scores showed little variation of EMT scores across space, intermediate scoring samples showed patches of high-scoring cells (Fig. 5e, segmented MERFISH data), suggesting a spatially determined component.

We partitioned the samples across three spatial EMT phenotypes—EMT-low, EMT-patched and EMT-high—and identified genes that were differentially expressed between malignant cells in tumors from the three spatial EMT phenotypes (Fig. 5f). EMT-patched and EMT-high phenotypes were each characterized by distinct cell cycle genes (EMT-patched: *CCND1*, *RB1* and *NF1*; EMT-high: *CDC20*); EMT-low samples were further characterized by *AGR2*, a potential biomarker of poor prognosis^53,54^. The differential expression changes between EMT-patched and EMT-high phenotypes were largely congruent across MERFISH, Slide-seq and sc/snRNA-seq but not CODEX or ExSeq (Fig. 5g,h).

**Fig. 6:**
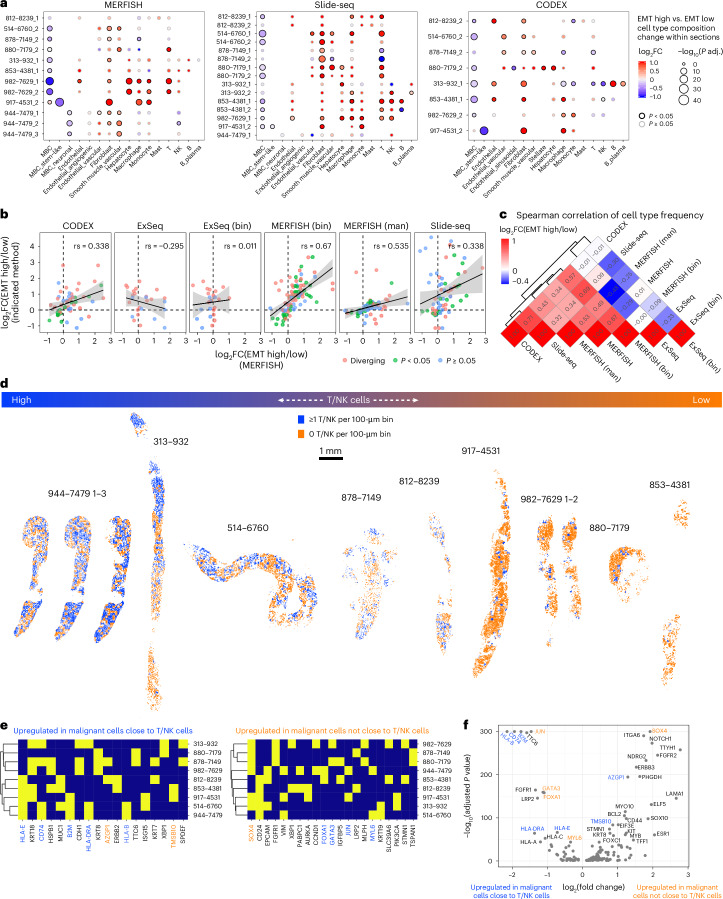
Characterizing the cellular neighborhoods of malignant expression phenotypes across spatial expression profiling methods. **a**, Dot plots depicting the log fold change (color) and significance (size) of differences in cell type frequencies between EMT-high and EMT-low neighborhoods (100 × 100-μm bins) within each section for MERFISH, Slide-seq and CODEX. ExSeq data did not yield any significant results. Replicates (serial sections) of the same biopsy are denoted with ‘_1–3’. *P* values were calculated using a two-sided Wilcoxon test and Benjamini–Hochberg multiple testing correction. **b**, Scatter plot relating the log fold changes of cell type frequency between EMT-high and EMT-low neighborhoods within samples as detected in cell-segmented MERFISH to the corresponding cell type frequency changes detected in the other indicated methods. The significance of differential cell type frequencies was calculated by a two-sided Wilcoxon test and Benjamini–Hochberg correction. The Spearman correlation is indicated; error bands indicate standard error. **c**, Clustered heatmap depicting the pair-wise Spearman correlation of methods based on cell type frequency log fold changes between EMT-high and EMT-low neighborhoods within samples, defined as in Fig. 5e, related to **b**. **d**, Spatial scatter plots of the malignant cells within the cell-segmented MERFISH data where each cell is colored as to whether or not it resides in the same 100 × 100-μm bin as at least one T/NK cell. **e**, Clustered binary heatmaps of whether or not a gene is among the top 10 differentially expressed genes between malignant cells residing close to a T/NK cell and those that do not within each biopsy, measured by cell-segmented MERFISH. Only genes that occur in at least two samples are shown. Genes are colored by their directionality in the common differential expression analysis. Genes with different directionality between patient-specific and combined analysis show discordant coloring. **f**, Volcano plot of differential gene expression analysis (two-sided Wilcoxon test, Benjamini–Hochberg correction) between malignant cells residing close to a T/NK cell and those that do not across all biopsies, measured by cell-segmented MERFISH data. Genes are colored by their directionality in the sample-specific differential expression analysis. Genes with different directionality between patient-specific and combined analysis show discordant coloring. FC, fold change; man, manual.

EMT-high (> sample median) and EMT-low (< sample median) local neighborhoods (100 × 100-μm bins) showed differences in cell type composition (Fig. 6a). Across all samples and methods (except ExSeq—no significant enrichments), malignant cells were depleted and fibroblasts were enriched in EMT-high neighborhoods (Fig. 6a). Interestingly, in EMT-high neighborhoods of sample 917 (the one sample with stem-like and non-stem-like malignant cells), stem-like malignant cells were depleted and non-stem-like malignant cells were slightly enriched (Fig. 6a; MERFISH and CODEX but not Slide-seq). Myeloid and lymphoid cell types showed mostly sample-specific enrichments (Fig. 6a). Overall, replicate sections (Fig. 6a) and all methods except ExSeq showed relatively good agreement (0.32 < *ρ* < 0.68) in terms of cell type composition differences between EMT-low and EMT-high neighborhoods (Fig. 6b,c).

To recover spatial patterns related to interactions between malignant and lymphoid cells, we tested if differences in malignant cell expression profiles are associated with differences in their proximity to T/NK cells (Methods). T/NK^+^ 100 × 100-μm bins generally formed patches, regardless of the overall level of T/NK infiltration (Fig. 6d). Malignant cells in T/NK^+^ bins showed higher expression of MHC-I and MHC-II genes (*HLA-E*, *CD74*, *B2M*, *HLA-DRA* and *HLA-B*), as expected, but also luminal epithelial genes (*KRT8*, *KRT18* and *MUC1*) and *ISG15* (Fig. 6e). On the other hand, genes upregulated in malignant cells in the T/NK^−^ bins included *SOX4* (in six of nine biopsies), consistent with the association of SOX4 expression with lower CD8^+^ T cell infiltration in primary TNBC^55^. Thus, *SOX4-*expressing malignant cells that seemingly avoid T/NK contact coexist in the same biopsies with malignant cells that engage in T/NK cell interactions. These patterns were also observed when analyzing malignant cells across all metastases jointly (Fig. 6f), as were additional key genes (for example, *GATA3* and *FOXA1* in T/NK^+^ regions; *TMSB10* and *AZGP1* in T/NK^−^ regions) that were recovered in different categories compared to the patient-specific analysis. Thus, although combining different biopsies can increase the power to detect common signals, patient-specific signals might be lost or even interpreted inversely.

## Discussion

We generated an integrated atlas of MBC based on single-cell and spatial expression profiling of 67 core needle biopsies from 60 patients. Spanning the clinical and molecular heterogeneity of MBC and incorporating a careful experimental design that enables comparison across methods provide opportunities for advances across BC research as well as method and algorithm development. This breadth-centered approach limits the statistical power for analyses of clinicopathologic subsets, and unique aspects of individual methods could not always be represented, including ExSeq’s nanometer resolution and Slide-seq’s potential for decomposed analysis. Nevertheless, in addition to providing insight into the architecture of MBC—including cell types, expression programs and their spatial relationships—and practical comparison across methods, we also leveraged the dataset to explore sources of heterogeneity and spatial expression phenotypes.

On a technical level, profiling method contributed to observed expression variability, including in key genes such as *ESR1* and *TRPS1*, a finding with implications for marker gene-based approaches. Among single-cell methods, snRNA-seq not only captured epithelial and stromal cells more efficiently but also more closely matched spatial data. ComBat performed well for platform correction on a pseudobulk level, and Harmony integrated the data well at the single-cell level.

Spatial profiling methods generally showed high agreement, and all recovered co-localization patterns within their profiling areas. ExSeq diverged the most from other methods, although local cell type frequencies were still similar. MERFISH performed particularly favorably in terms of separable, single-cell molecular profiles and faithfully recovered patient-specific expression signals as the primary driver of malignant, but not non-malignant, cell-intrinsic variability.

The malignant compartment was characterized by substantial inter-patient heterogeneity but still revealed intriguing patterns: basal-like biopsies formed a highly correlated exclusive subcluster; EMT programs were robust among single-cell methods and demonstrated inter-patient and intra-patient heterogeneity in three spatial phenotypes, complementing prior studies of EMT marker expression heterogeneity both within primary BC^56^ and between matched primary and metastatic biopsies^57^; and patient-specific CNA profiles and expression programs were maintained across time, site and even changes in receptor subtypes, in contrast to prior orthogonal studies of genomic evolution and diversity through disease progression and metastasis^58–60^.

In the immune compartment, macrophages were the most frequent cell type, although their frequency was influenced by the most recent treatment class and specifically increased with prior immunotherapy. Across methods, we identified two macrophage states characterized by *CD163*/*CD68*/*APOE*/*HIF1A* and *MKI67*, respectively. Although *APOE* expression was reported to promote T cell effector functions^61^, we did not find a significant spatial correlation between expression of *APOE* in macrophages and *PDCD1* or *CTLA4* in T/NK cells. While macrophages were ubiquitous, they weakly avoided malignant cells; T/NK cells showed more variable infiltration levels. Notably, T/NK localization relative to malignant cells was associated with expression patterns in malignant cells— co-localization with higher expression of MHC components; exclusion with increased *SOX4*—expanding on previous studies linking SOX4 expression to immune evasion in primary TNBC^55^. Future work will further investigate the molecular underpinnings of these cell states and spatial interactions and their translational significance.

## Methods

### Ethics statement

All samples included in this study were voluntarily donated by patients who provided informed consent under an institutional review board (IRB)-approved protocol (DF/HCC no. 05-246), which includes permission for sample acquisition, clinical data abstraction, sample analysis and data sharing. Analysis of biospecimens at the Broad Institute was performed under Broad Institute protocol number 15-370B.

### Sample acquisition, handling and annotation

Tissues were collected as described in detail previously^15^. Clinical annotations were generated from the electronic medical record under the supervision of a board-certified medical oncologist and a cancer registrar following HTAN clinical data standards (https://humantumoratlas.org/standard/clinical), which are based on the National Cancer Institute Genomic Data Commons model (https://gdc.cancer.gov/about-data/gdc-data-processing/clinical-data-standardization).

For snRNA-seq and spatial expression assays, core needle biopsies were either snap frozen or frozen in optimal cutting temperature (OCT) compound (Tissue-Tek, Sakura) to preserve. Cores were pre-coated with OCT by putting a thin layer of OCT down in the cryomold before placing an individual core in the center of the OCT mold in a straight line and adding additional OCT to fill the cryomold. The cryomold was then placed on dry ice for 5–15 min until the block was opaque before storing it at −80 °C. For scRNA-seq, core needle biopsies were transferred from interventional radiology into DMEM medium and processed upon arrival at the Broad Institute.

### Generation of snRNA-seq data

snRNA-seq was performed as described previously^15^. Specifically, frozen tissue was placed on ice and in one well of a plate (STEMCELL Technologies, 38015), and 1 ml of TST buffer was added to the well. Tissue was kept on ice and cut into pieces with Noyes spring scissors (Fine Science Tools, 15514-12) for 10 min. Tissue mixture was filtered through a 40-μm Falcon cell strainer (Thermo Fisher Scientific, 08-771-1). The well was washed and filtered with 1 ml of detergent buffer solution, and 3 ml of 1× ST buffer was added to a total well volume of 5 ml. The solution was centrifuged in a 15-ml Eppendorf tube for 5 min at 500*g* and 4 °C in a swinging bucket centrifuge. Pellet was resuspended in 1× ST buffer with a resuspension volume of 100–200 μl based on pellet size. The single-nucleus suspension was filtered through a 35-μm Falcon cell strainer (Corning, 352235). In total, 8,000 (V3) or 10,000 (V2) nuclei were selected with a C-chip disposable hemocytometer (VWR, 82030-468) and transferred to Chromium chips for the Chromium Single Cell 3′ Library (V2 or V3) per the manufacturer’s instructions (10x Genomics).

### Generation of scRNA-seq data

scRNA-seq was performed as described previously^15^. Specifically, samples were washed in cold PBS and transferred into a 2-ml Eppendorf tube containing dissociation mixture (950-µl volume of RPMI 1640 (Thermo Fisher Scientific, 11875093) + 10 µl of 10 mg ml^−1^ DNAse I (Sigma Aldrich, 11284932001) + 40 µl of 2.5 mg ml^−1^ Liberase (Sigma Aldrich, 5401127001)). Next, the sample was minced in the Eppendorf tube using spring scissors (Fine Science Tools, 15514-12) into fragments less than approximately 0.4 mm and incubated at 37 °C while rotating horizontally at approximately 14 r.p.m. for 10 min, followed by pipetting the sample 20 times with a 1-ml pipette tip at room temperature. The incubation and pipetting were repeated a second time before transfer to a 1.7-ml Eppendorf tube and centrifugation at 300–580*g* for 4–7 min at 4 °C. The pellet was then resuspended in 200–500 µl of ammonium–chloride–potassium (ACK) RBC lysis buffer (Thermo Fisher Scientific, A1049201) and incubated for 1 min on ice, followed by the addition of cold PBS at twice the volume of the ACK. The cells were pelleted by a short centrifugation for 8 s at 4 °C using the short spin setting with centrifugal force ramping up to, but not exceeding, 11,000*g*. This procedure was repeated up to three times until the pellet was no longer red or pink. To remove cell clumps, the pellet was resuspended in 100 µl of TrypLE (Life Technologies, 12604013) and incubated while constantly pipetting at room temperature for 1 min with a 200-µl pipette tip. TrypLE was inactivated by adding 200 µl of cold RPMI 1640 with 10% FBS. The cells were pelleted using short centrifugation as described above. The pellet was resuspended in 50 µl of 0.4% BSA (Ambion, AM2616) in PBS. To assess the single-cell suspension, viability and cell count, 5 µl of Trypan blue (Thermo Fisher Scientific, T10282) was mixed with 5 µl of the sample and loaded onto an INCYTO C-Chip Disposable Hemocytometer, Neubauer Improved (VWR, 82030-468). The cell concentration was adjusted if necessary to a range of 200–2,000 cells per microliter. A total of 8,000 cells were loaded into each channel of the 10x Genomics Single Cell Chromium Controller for the Chromium Single Cell 3′ Library (V2 or V3) per the manufacturer’s instructions (10x Genomics).

### 10x library generation and sequencing

Single cells and nuclei were partitioned into droplets with gel beads in the Chromium Controller. After emulsions were formed, barcoded reverse transcription of RNA took place. This was followed by cDNA amplification, fragmentation and adapter and sample index attachment, all according to the manufacturer’s recommendations. Libraries from four 10x channels were pooled together and sequenced on one lane of an Illumina HiSeq X, or on one flow cell of a NextSeq, with paired-end reads as follows: read 1, 26 nt; read 2, 55 nt; index 1, 8 nt; index 2, 0 nt.

### Processing and quality assurance of the sc/snRNA-seq data

Raw sequencing reads were processed using the cellranger_cellbender_workflow snapshot 6 on TERRA (https://app.terra.bio/), using the human genome GRCh38 as reference and retaining intronic reads for snRNA-seq but not for scRNA-seq. This workflow featured Cell Ranger version 3.0.2 and Cell Bender version 0.1.0. An initial processing of the resulting count matrices, including quality assessment and automated cell type annotation (see below), and doublet detection with scrublet version 0.2.1 was performed individually for each sample using Seurat version 3.1.1^62^).

Quality filtering was performed simultaneously on all samples, once all samples had been obtained and processed, to obtain data-driven quality filtering thresholds to account for biological and technical differences between samples. For example, immune cells that tend to contain less RNA than malignant cells were filtered with more lenient thresholds.

Following this rationale, low-quality cells were filtered out based on low or extremely high unique molecular identifier (UMI) counts, low gene counts and high mitochondrial read contributions in a manner dependent on cell type, protocol and chemistry (V2/V3).

The following algorithm was used to determine the thresholds for each filter group:

High threshold filter: mitochondrial genes <50%, number of genes <8,000, number of UMIs <20,000.

Low threshold filter for genes per cell: If the median number of genes per cell in the filter group of a given cell is >1,300, then cells with >700 genes are retained; if the group median is <1,300 and >600, then cells with >300 genes are retained; if the group median is <600, then cells with >100 genes are retained.

Low threshold filter for UMIs per cell: If the median number of UMIs per cell in the filter group of a given cell is >1,800, then cells with >1,100 UMIs are retained; if the group median is <1,800 and >900, then cells with >600 UMIs are retained; if the group median is <900, then cells with >300 UMIs are retained.

Samples with extremely low numbers of recovered cells were excluded as failed.

### Cell type annotation in sc/snRNA-seq

In an initial automated and sample-wise annotation, cells were annotated using the R package SingleR version 1.0.3 (https://bioconductor.org/packages/release/bioc/html/SingleR.html) with both its built-in reference datasets (HPCA and Blueprint) in a cell-wise and cluster-wise annotation scheme, and annotations were then refined by harmonizing labels across the reference dataset and within clusters.

After combining all snRNA-seq or scRNA-seq samples into one anndata object each, as well as joint processing using the SCANPY version 1.7.2 workflow, including normalization, log1p transformation, scaling, highly variable gene selection, regression of total counts and mitochondrial counts, principal component analysis (PCA), nearest neighbor finding, Leiden clustering and two-dimensional (2D) projection using uniform manifold approximation and projection (UMAP), the initial automated annotation was further refined using the context of all sc/snRNA-seq samples, respectively.

Single cells that were annotated with a cell type label that was not compatible with their cluster’s annotation were removed as unreliable. Clearly distinct clusters that were annotated with the same cell type label were investigated in detail using marker genes and assigned more specific cell type labels. For a simplified annotation, all cells then received a second label based on their cell type label to be assigned to one of the four compartments: malignant, stromal, myeloid and lymphoid.

### CNA in the sc/snRNA-seq data

CNAs in the sc/snRNA-seq were scored using InferCNV version 1.2.0 (https://github.com/broadinstitute/inferCNV). Sample-wise analysis was performed by assigning the following cell types as normal reference—T cells, NK cells, monocytes, macrophages, fibroblasts and endothelial cells—and calling CNAs in all other cell types. In particular, we did not include hepatocytes as reference cells because they are known to be polyploid and B/plasma cells because of disproportionately high expression of certain genes related to antibody production.

The cross-sample combined analysis was performed by selecting normal (non-malignant) reference cells across all samples in an even manner and calling CNAs in all malignant cells across all samples separately for the snRNA-seq and scRNA-seq data. InferCNV’s built-in CNA heatmap was then assessed for interesting patterns and used for presentation.

### Variance analysis in the sc/snRNA-seq data

Variance analysis in the sc/snRNA-seq data was performed using the R package variancePartition version 1.14.0 (ref. ^63^), which uses linear mixed models to quantify variation in gene expression that can be attributed to different biological or technical variables (patient ID (individual), method (sc/sn), site, most recent treatment class, histology, metastatic presentation and receptor status). Apart from using this tool for the study of expression variability in pseudobulk data (average expression across all cells per sample and compartment), we also used it to assess variability in cell type composition. The rationale behind this approach is that both RNA-seq expression and cell type abundances are primarily count data that are normalized to represent the frequency or representation of one entity (gene or cell type) among all measurements. However, to account for stronger expected interdependence between cell types due to their lower number compared to genes (~20 versus ~20,000), we used Pearon’s contingency ratios^64^ instead of normalization by total counts as used for the expression variance analysis.

### De novo characterization of malignant expression programs using iNMF

To find de novo malignant expression programs in our sc/snRNA-seq across all samples, we used iNMF as implemented in the R package LIGER version 0.5.0.9000 (ref. ^30^), which identifies and separates common and sample-specific factors in high-dimensional single-cell data. We ran this analysis separately for snRNA-seq and scRNA-seq data, setting the k parameter to 20 to receive 20 expression programs and the lambda parameter to 40 to ensure sufficient integration and separation of sample-specific signals. These parameters were found empirically. The thus-obtained 40 expression programs were then correlated by pair-wise Pearson correlation based on the gene importance for the respective programs as represented in the feature matrix *W*. This way, we were able to identify corresponding programs in the sc/snRNA-seq data as highly correlated programs.

### Scoring of expression programs in sc/snRNA-seq and spatial data

Expression programs defined by specific sets of genes were performed using either Seurat’s version 3.1.1 or SCANPY’s version 1.7.2 built-in functions AddModuleScore or score_genes, respectively, with default parameters. Seurat was used to score the subcell-type marker genes^17^ as well as the hallmark gene sets in the Molecular Signatures Database (MSigDB)^65,66^, and SCANPY version 1.7.2 was used to score the scRNA-seq-derived iNMF EMT program genes (*IGFBP7*, *SPARC*, *COL1A2*, *COL4A1*, *COL3A1*, *BGN*, *ACTA2*, *FN1*, *COL4A2*, *TAGLN*, *DCN*, *COL1A1*, *LUM*, *COL6A3*, *POSTN*, *AEBP1*, *COL6A2*, *VIM*, *TIMP1*, *TPM2*, *COL5A1*, *CALD1*, *COL6A1*, *A2M*, *SPARCL1*, *THY1*, *VCAN*, *CCN2*, *GNG11*, *PDGFRB*, *RGS5*, *ITGA1*, *MYL9*, *COL5A2*, *COL18A1*, *THBS2*, *IGHA1*, *CAVIN1*, *ELN*, *NID1*, *LHFPL6*, *APOE*, *IGLC3*, *HSPG2*, *CAV1*, *TCF4*, *NNMT*, *ASPN*, *FSTL1* and *MGP*), of which 20 genes are represented in MERFISH and ExSeq (*TCF4*, *COL4A1*, *BGN*, *COL1A2*, *FN1*, *COL1A1*, *ACTA2*, *MYL9*, *HSPG2*, *TIMP1*, *VIM*, *THY1*, *APOE*, *COL3A1*, *DCN*, *LUM*, *TAGLN*, *TPM2*, *GNG11* and *COL4A2*) and three in CODEX (*VIM*, *THY1* and *COL4A2*). Scoring was performed on all samples profiled with a given method. The choice of which tool to use was based purely on the environment (R versus Python) that the respective analysis branches were performed in.

### Integration of sc/snRNA-seq data or spatial data on a pseudobulk or single-cell/bead/bin level

To compare malignant pseudobulk expression profiles, the pseudobulk expression matrix was corrected for profiling method effects using the ComBat function from the R package sva version 3.34.0 (ref. ^67^), with profiling method as batch variable and receptor status as well as biopsy site as covariates.

To integrate snRNA-seq and scRNA-seq data at the single-cell level, the function ‘harmonize’ from the Python package Harmony-pytorch version 0.1.4 (ref. ^37^) and SCANPY’s BBKN wrapper (external.pp.bbknn) based on the Python package BBKNN version 1.5.1 (ref. ^38^) were used. Each function was run with profiling method as batch variable and default parameters otherwise. After integration, Leiden clustering was performed using the SCANPY function ‘leiden’ with a resolution of 0.4. The integrated dataset was only used to demonstrate data integration but not for other analyses. (These methods do not correct the expression matrix but align the observations in a lower-dimensional space (Harmony: PCA; BBKNN: *k*-nearest neighbor graph)).

To analyze macrophage subsets in sc/snRNA-seq and spatial data, annotated macrophages were integrated separately for each measurement method using the function ‘harmonize’ from the Python package Harmony-pytorch version 0.1.4 with patient as batch variable and default parameters otherwise. After integration, Leiden clustering was performed using the SCANPY function ‘leiden’ with a resolution of 0.6. Small clusters expressing non-macrophage marker genes were detected in all methods and removed from further analysis, followed by re-intergation and re-clustering.

### PAM50 molecular subtype assignment

To assign research-based PAM50 subtypes, log2 + 1-transformed counts from the full (including all cell types) pseudobulk data were rescaled relative to those of a receptor status-balanced version of this cohort, in which samples were resampled to achieve the ER^+^ to ER^−^ receptor status ratio in the UNC training set, from which the PAM50 subtype centroids were derived^68,69^. The R package genefu version 2.20.0 (ref. ^70^) was used to call research-based PAM50 subtypes using the rescaled expression values and Spearman correlation to the PAM50 subtype centroids. Samples with a PAM50 centroid correlation less than 0.10 for each centroid were not assigned a PAM50 subtype.

### Sectioning for spatial expression profiling and H&E staining

The tissue OCT blocks were acclimated to −20 °C inside the cryostat (Leica, CM1950) for 30 min before sectioning at 10-μm thickness. Serial sections were placed on the required glass slides for each of the methods used. Sections were placed such that the same region of interest could be assessed across all methods.

### H&E staining and histopathological annotation

A slide adjacent to the experimental slides was stained for H&E with standard histology techniques. H&E slides were scanned on an Aperio Pathology AT2 Slide Scanner (Leica) using ×20 magnification. Each H&E slide was reviewed by a board-certified pathologist (S.J.R.) for QC assessment and annotated to indicate the location of tumor regions using standard pathological criteria. This review was conducted with a traditional bright-field microscope and included assessment of preservation of tissue integrity and morphology after freezing and OCT embedding, evaluation of tissue viability, assessment of tumor content and fibrotic tissue content and scoring for inflammation on a 0–3 scale. Samples that failed this QC step (9/25 samples) exhibited either very low sample viability (<2% viable cells) or extensive tissue damage or had less than 5% tumor content.

### Slide-seq data generation

To generate Slide-seq data, the Slide-seq puck was placed on a microscope glass slide with the beads facing upwards and held in place with a drop of water between the glass slide and the puck coverslip. By turning the microscope glass slide upside down, the puck surface was aimed at the region of interest in the tissue section by lowering the puck over the tissue section and allowing a quick melting of tissue and puck to occur before removing the puck:tissue sandwich outside the cryostat. The puck was moved with forceps to an Eppendorf tube pre-filled with 200 μl of hybridization buffer (6× SSC with 2 U μl^−1^ RNase inhibitor (Lucigen, 30281)) and incubated for 15 min at room temperature. A wash followed hybridization by dipping the puck once into 1× Maxima RT buffer. First-strand cDNA synthesis was performed by placing the puck in 200 μl of first-strand synthesis mixture (1× Maxima RT buffer, 1 mM of each dNTP, 0.05 U μl^−1^ RNase inhibitor (Lucigen, 30281), 2.5 μM template switch oligonucleotide (Integrated DNA Technologies (IDT), 5′-AAGCAGTGGTATCAACGCAGAGTGAATrG+GrG-3′) and 10 U μl^−1^ Maxima H Minus Reverse Transcriptase (Thermo Fisher Scientific, EP0742)) and incubated at room temperature for 30 min followed by 52 °C for 90 min.

Tissue digestion was thereafter performed by the addition of 200 μl of 2× tissue digestion mix (200 mM Tris-Cl pH 7.5, 400 mM NaCl, 4% SDS, 10 mM EDTA) with 1:50 proteinase K (New England BioLabs (NEB), P8107S) to the first-strand reaction mixture with gentle pipette mixing and incubation at 37 °C for 30 min.

After the addition of 200 μl of wash buffer (10 mM Tris pH 8.0, 1 mM EDTA, 0.01% Tween 20) to the tissue digestion mixture, the puck beads were removed from the coverslip surface and released into suspension by vigorously pipetting, and the glass was discarded. The beads were pelleted by centrifugation at 3,000*g* for 2 min, and the supernatant was removed. The bead pellet was washed in 200 μl of wash buffer and centrifuged as before for a total of three washes, followed by a final wash in 10 mM Tris-HCl, pH 7. Subsequent exonuclease treatment was performed by resuspension of the bead pellet in 200 μl of Exonuclease I reaction mixture (1× ExoI buffer with 10 U μl^−1^ Exonuclease I (NEB, M0293L)) and incubated at 37 °C for 50 min, followed by one wash with 200 μl of wash buffer added directly to the exonuclease mixture. After supernatant removal, the wash step was repeated twice for a total of three washes. The bead pellet was resuspended in 200 μl of freshly prepared 0.1 N NaOH and incubated for 5 min at room temperature. After the addition of 200 μl of wash buffer, the beads were centrifuged for 2 min at 3,000*g*, and the wash was repeated a total of three times.

Second-strand synthesis was performed by the addition of 200 μl (1× Maxima RT buffer, 1 mM of each dNTP, 10 μM dN-SMRT oligonucleotide (IDT, 5′-AAGCAGTGGTATCAACGCAGAGTGANNNGGNNNB-3′) and 0.125 U μl^−1^ Klenow enzyme (NEB, M0210)) to the bead pellet and incubation at 37 °C for 1 h. Thereafter, 200 μl of wash buffer was added to the mixture and centrifuged for 2 min at 3,000*g*. The wash was repeated a total of three times, followed by a final wash in RNase/DNase-free water. The bead pellet was resuspended in 50 μl of PCR mix (1× Terra Direct PCR mix buffer, 2 µl Terra polymerase (Takara, 639270), 2 μM TruSeq PCR handle primer (IDT, 5′-CTACACGACGCTCTTCCGATCT-3′) and 2 μM SMART PCR primer (IDT, 5′-AAGCAGTGGTATCAACGCAGAGT-3′)). PCR was performed with the following program: 98 °C for 2 min; four cycles of 98 °C for 20 s, 65 °C for 45 s and 72 °C for 3 min; 11 cycles of 98 °C for 20 s, 67 °C for 20 s and 72 °C for 3 min; 72 °C for 5 min; hold at 4 °C.

The cDNA was incubated with 0.6× volumes of AMPure XP beads for 10 min at room temperature. The AMPure XP beads were then pelleted using a magnetic separator for 5 min, followed by two washes with 80% ethanol for 30 s each, and the cDNA was eluted with 50 μl of EB solution. The bead purification was repeated at a 0.6× volume of AMPure XP beads:cDNA with two washes with 80% ethanol and final elution with 12 μl of EB. The size and concentration of the final cDNA were assessed on a Bioanalyzer high-sensitivity DNA chip (Agilent, 5067-4626) and on a Qubit high-sensitivity dsDNA kit (Invitrogen, Q32851), respectively. Thereafter, 600 pg of cDNA was tagmented with a Nextera XT kit (Illumina, FC-131-1096) according to the manufacturer’s instructions. The libraries were indexed with PCR amplification with TruSeq5 (IDT, 5′- AATGATACGGCGACCACCGAGATCTACACTCTTTCCCTACACGACGCTCTTCCGATCT-3′) and the N700 series barcoded index primers and the following PCR program: 72 °C for 3 min; 95 °C for 30 s; 12 cycles of 95 °C for 10 s, 55 °C for 30 s, 72 °C for 30 s and 72 °C for 5 min; hold at 4 °C.

Final purification of the DNA with AMPure XP beads at a 0.6:1 volume ratio of beads:DNA and elution with 12 μl of EB yielded sequencing-ready libraries. The library concentrations were diluted to 4 nM each, and three Slide-seq samples were pooled together. The samples were sequenced at a 1.8 pM concentration on an Illumina NextSeq high-output flow cell with the following settings: read1, 44 bases; read2, 39 bases; and index1, 8 bases.

Raw data were processed using the Slide-seq pipeline (https://github.com/MacoskoLab/slideseq-tools).

The quality of all samples was evaluated, and samples with an average read count per bead lower than 150 as well as those with an unrecognizable shape (which prevented spatial alignment) were excluded from further analysis.

### CODEX data generation

CODEX data generation was performed as described previously without major adjustment for the MBC tissue^7,8^. The detailed protocol is available on https://www.protocols.io/ (ref. ^71^). Specifically, antibody panels for CODEX imaging were chosen to include targets that would be anticipated to identify MBC as well as cells of the innate and adaptive immune system. Each antibody was conjugated to a unique oligonucleotide barcode. Detailed panel information can be found in Supplementary Table 5. For panel validation, antibody–oligonucleotide conjugates were tested in low-plex fluorescence assays. Staining patterns were compared against the expected patterns already established for immunohistochemistry within positive control tissues of the human tonsil. Staining patterns were also compared against H&E morphology staining to confirm the location of the markers. Signal-to-noise ratio was also evaluated at this step. Antibody–oligonucleotide conjugates were then tested altogether in a single CODEX multicycle.

CODEX multiplexed imaging was executed according to the previously described protocols and imaging setup and instructions for CODEX staining of frozen specimens from Akoya Biosciences. In brief, after the sample acquisition and OCT embedding, 7-µm sections were cut in a cryostat after OCT blocks were equilibrated to the cryostat temperature for at least 30–40 min. Tissue sections were dragged over the surface of cold poly-l-lysine-coated coverslips and spread inside the cryostat by transiently warming up the bottom surface of the coverslip with a finger. Before staining, the sections removed from the freezer were dried for 5 min on the surface of Drierite. Dried coverslips with sections on them were dipped for 10 min into room temperature acetone and then fully dried for 10 min at room temperature. Sections were then rehydrated for 5 min in S1 (5 mM EDTA (Sigma-Aldrich)), 0.5% w/v BSA (Sigma-Aldrich and 0.02% w/v NaN_3_ (Sigma-Aldrich) in PBS (Thermo Fisher Scientific)) and further re-fixed for 20 min at room temperature in S1 with 1.6% formaldehyde. Formaldehyde was washed off twice with S1, and sections were equilibrated in S2 (61 mM NaH_2_PO_4_ ∙ 7 H_2_O (Sigma-Aldrich), 39 mM NaH_2_PO_4_ (Sigma-Aldrich) and 250 mM NaCl (Sigma-Aldrich) in a 1:0.7 v/v solution of S1 and double-distilled water (ddH_2_O); final pH 6.8–7.0) for 10 min and blocked in blocking buffer (ref. ^2^) for 30 min. All steps to follow were exactly as in Black et al.^8^ or the Akoya CODEX instructions—this entails cyclic stripping, annealing and imaging of fluorescently labeled oligonucleotides complementary to the oligonucleotide on the conjugate.

Automated image acquisition and fluidics exchange were performed using an Akoya CODEX instrument driven by CODEX driver software (Akoya Biosciences) and a Keyence BZ-X710 fluorescence microscope configured with four fluorescent channels (DAPI, FITC, Cy3 and Cy5) and equipped with a CFI Plan Apo λ ×20/0.75 objective (Nikon). Hoechst nuclear stain (1:3,000 final concentration) was imaged in each cycle at an exposure time of 1/175 s. Biotinylated CD39 (clone A1, Biolegend) was used at a dilution of 1:500 and visualized in the last imaging cycle using DNA streptavidin-PE (1:2,500 final concentration). DRAQ5 nuclear stain (1:500 final concentration) was added and visualized in the last imaging cycle. Each tissue was imaged with a ×20 objective in a 7 × 9 tiled acquisition at 1,386 × 1,008 pixels per tile and 396-nm-per-pixel resolution and 13 *z*-planes per tile (axial resolution, 1,500 nm). Images were subjected to deconvolution to remove out-of-focus light.

Raw imaging data were processed using the CODEX Uploader (https://github.com/nolanlab/CODEX) for image stitching, drift compensation, deconvolution and cycle concatenation. Processed data were then segmented using CellVisionSegmenter, an open-source, pre-trained nucleus segmentation and signal quantification software based on the Mask region-convolutional neural network (R-CNN) architecture. CellVisionSegmenter was trained on manually annotated CODEX multiplexed imaging data and can successfully segment both dense and diffuse cellular tissues (https://github.com/bmyury/CellVisionSegmenter; https://github.com/michaellee1/CellSeg)^68^. As such, only one parameter was altered for the segmentation of the HTAPP dataset: the growth pixels of the nuclear mask. This was experimentally determined to be optimal at a value of 3. After the upload, the images were visualized in ImageJ (https://imagej.net/) and re-evaluated for specific signal. Any markers that produced a low signal-to-noise ratio or an untenable pattern were excluded from the ensuing analysis. Finally, all samples were manually checked for presence of obvious signs of unexpected signal appearance or distribution indicative of device or protocol error. None was detected, and all samples were considered fit for downstream image analysis.

### Gene panel design for MERFISH and ExSeq

To select a set of genes for spatial profiling of MBC biopsies with MERFISH and targeted ExSEQ assays, we developed a ‘collect-and-filter’ approach to allow flexibility in the final number of selected genes. First, a preliminary list of 510 potentially relevant genes was assembled (collected) based on prior knowledge and literature as well as on our MBC sc/snRNA-seq data. Genes were chosen to represent various aspects of BC biology, metastasis and the tumor immune microenvironment as well as cell types and programs discovered from sc/snRNA-seq. The preliminary list was then filtered down to 300 genes (the experimental size of the panel) based on expression statistics as measured in the MBC scRNA-seq dataset and manual priority (0–1) assignment. During probe design, three of the selected 300 genes were excluded as they did not meet technical criteria (all three transcripts were too short), reducing the final gene set to 297 genes. Below, we describe in more detail the initial selection of 510 genes and their filtering down to 300.

#### Gene collection

To generate a preliminary list of genes likely to be broadly relevant for characterization of cell types and programs in MBC lesions, we pursued three broad criteria: (1) prior knowledge based on expertise and relevant scientific publications; (2) genes coding for proteins targeted in CODEX proteomic assays also applied to the same MBC HTAPP tumor samples; and (3) genes representing cell types and programs from preliminary sc/snRNA-seq data from 21 MBC biopsies.

The prior knowledge-driven gene selection (1) started by identifying categories of genes known to be important in MBC and in cancer in general and reviewing available literature to select representative genes of each category:

- Canonical cell-type-specific markers (for example, EPCAM for epithelial cells, CD19 for B cells, CD4 for T helper cells, CD8 for cytotoxic T lymphocytes, CD56 for NK cells and CD14 for macrophages)
- Clinical breast cancer biomarkers (for example, ESR1, PGR and ERBB2)
- Breast cancer intrinsic subtypes^72,73^
- Hallmarks of cancer: evasion of apoptosis, for example, BCL2; EMT, for example, VIM; immune evasion, for example, CD274; senescence, for example, TP53; proliferation, for example, MKI67, etc.^71,72^
- Epithelial hierarchy in the normal breast^74–77^
- ER signaling^78^
- Genomic landscape of MBC and therapeutic resistance^59,79–83^

The pre-defined CODEX target genes were included in the panel to ensure congruence and subsequent integration with matching CODEX data. To this end, we translated protein identifiers to gene identifiers and assigned the resulting genes priority 1 to be included in the panel (see the ‘Gene filtering’ subsection).

The data-driven gene selection was performed on the sc/snRNA-seq data available at that time using Seurat version 2.3.4. The data used for gene selection consisted of 21 MBC samples (six snRNA-seq, 15 scRNA-seq) and represent only a subset of the final dataset of 37 snRNA-seq and 30 scRNA-seq. Single-cell profiles with fewer than 500 genes and single-nucleus profiles with fewer than 200 genes were removed. Preliminary cell types were annotated using the R package SingleR version 1.0.1 (https://bioconductor.org/packages/release/bioc/html/SingleR.html) in single-cell mode with the built-in HPCA reference and standard parameters. To identify cell-type-specific genes—that is, genes with high cell type predictive power—we trained a support vector machine (SVM) classifier (R package liblineaR version 2.10-8) and used the assigned feature weights to select highly predictive genes for each cell type. Data were downsampled to 200 randomly selected cells per cell type to ensure class balance, and predictive power of the classifier was assessed through five-fold cross-validation and prediction accuracy. In a first pass, baseline accuracy was determined by training and testing a classifier on all variable genes. In a second pass, per cell type, only genes with a ranked cumulative relative weight below 0.4 (single-nucleus data) and 0.45 (single-cell data) (that is, all top weighted genes that together account for 40% or 45% of relative weight, respectively) were used to train a second classifier on a second independently downsampled dataset with the same specifications. Again, accuracy was assessed in five-fold cross-validation and compared to baseline accuracy to ensure that, by reducing the number of genes and using a different subset of the data, accuracy was not significantly reduced. Additionally, we also determined the classification error rate using a random forest classifier (R package randomForest version 4.6-14) to confirm that the observed good performance was not classifier dependent. Of the thus-selected genes, all genes with a ranked cumulative relative weight below 0.3 (single-nucleus data) or 0.35 (single-cell data) were assigned priority 1, whereas the remaining genes were assigned priorities lower than 1 based on their relative total weights across all cell types (see the ‘Gene filtering’ subsection).

To represent the BC-intrinsic subtypes, the PAM50 subtype-defining genes^64^ were refined using a similar approach as the one described above based on the single-cell and single-nucleus data. In a first pass, all 50 PAM50 genes were used to detect baseline accuracy of discriminating the PAM50 subtypes, and, in a second pass, all genes with a ranked cumulative relative weight lower than 0.8 (single-cell and single-nucleus data) were used to determine classification accuracy and assigned priority 1, whereas the remaining genes were assigned priorities lower than 1 based on their relative total weights across all cell types (see the ‘Gene filtering’ subsection).

To select genes that represent cellular programs within cell types, we applied topic modeling separately on the major cell types present in the single-cell dataset (malignant cells, T cells, NK cells, fibroblasts, endothelial cells, monocytes/macrophages/dendritic cells, B cells and plasma cells). We used the FitGoM() function of the R package CountClust version 1.12.0 to fit a grade of membership (GoM) model to the raw count data of up to 4,000 randomly sampled cells per cell type. The tolerance value of the GoM model was set to 0.01 for all cell types. The number of topics (*K*) to be fitted was empirically determined for each cell type by fitting models with a range of sensible values for *K* and comparing the Bayesian information criterion (BIC) of the different models. For each cell type, *K* was selected to be greater than or equal to 3 and to represent a local minimum in BIC. Finally, separate models were fit for each of the following cell types with the indicated parameters after excluding ribosomal and mitochondrial genes: malignant cells (*K* = 13), T cells (*K* = 3), NK cells (*K* = 3), fibroblasts (*K* = 4), endothelial cells (*K* = 5), monocytes/macrophages/dendritic cells (*K* = 7), B cells (*K* = 3) and plasma cells (*K* = 10). For each topic, the top 30 genes were identified using the function ExtractTopFeatures() and subjected to GSEA using enrichR version 1.0^83^ querying the GO_Biological_Process_2018 database. Topic loadings across cells as well as Gene Ontology (GO) terms enriched with an adjusted *P* value false discovery rate (FDR) < 0.05 were manually inspected for interesting patterns. Of the genes defining topics and GO terms deemed interesting, the gene with the highest loading for each topic was assigned priority 1, whereas the other genes were assigned priority 0 (see the ‘Gene filtering’ subsection).

#### Gene filtering

To select 300 genes from the list of 510 assembled through the different approaches described above, we devised a filtering strategy to make sure that genes are expressed in, and are variable across, the single-cell expression dataset while preserving the diversity of cellular and biomedical aspects represented by the 510 genes and summarized as nine categories and 83 selection types of genes. A gene was included under the following conditions: (mean normalized expression > 0.15 OR variability > 0.025 OR number of categories > 1) AND (mean normalized expression > 1.5 and < 4 OR variability > 0.25 OR priority = 1 OR number of categories > 1) with variability defined as the fraction of cells with an absolute scaled expression value greater than 1 across all cells and mean normalized expression calculated across all cells of the highest expressing cell type or epithelial (malignant) cells in the case of genes selected due to their known relevance in MBC. Three genes were identified as being too short during the probe generation step, because they did not have sufficient length to accommodate the placement of a sufficient number of unique probes. The total number of genes assessed was, thus, 297, representing all nine categories and 82 of the 83 original gene types (Supplementary Table 3). This high retention rate of represented gene types confirmed that we were still covering all major cell types, subtypes and programs of interest with the reduced gene set and allowed us to confidently move forward with it.

### MERFISH data generation

The detailed protocol for MERFISH data generation is available on https://www.protocols.io/ (ref. ^84^). The MERFISH protocol was divided into three parts: probe design/generation, tissue processing and imaging and analysis/segmentation.

In addition to the 297 genes selected for MERFISH as described above, two additional genes, *ALB* and *LIPE*, were added to the gene panel for ready identification of the common host tissue cell types found in liver (hepatocytes) and adipose (adipocytes) tissues, respectively. For design and construction of encoding probes, each of the 291 genes imaged in the combinatorial imaging rounds was assigned to a unique binary barcode drawn from a 22-bit, Hamming distance 4, Hamming weight 4 encoding scheme. Ninety-four extra ‘blank’ barcodes that were not assigned to any genes were included to provide a measure of the false-positive rate. Each bit of the 22-bit code was associated with a unique readout sequence, and, for each gene, the readouts corresponded to the four ‘on-bit’ (bits that read ‘1’) of the gene’s assigned barcode. For each gene, 60 encoding probes were generated, comprising a 30-mer target sequence, three readout sequences corresponding to the gene and PCR primer sequences for library amplification. Template DNA for the encoding probes used for the 291 multiplexed genes was synthesized as a complex oligo pool and used to construct the final MERFISH probe set, as described previously^85^. Encoding probes for the eight genes measured as sequential single-molecule FISH (smFISH) rounds were designed in a similar fashion as described above, except: (1) 48 probes were generated for each gene; (2) one unique readout sequence was used for each gene; and (3) PCR primers were omitted. Encoding probes were then synthesized in a 96-well plate format and mixed to suitable final concentration.

Sliced samples were placed on poly-d-lysine-coated coverslips, fixed with 4% formaldehyde, permeabilized in 70% ethanol, photobleached with white light and then hybridized with the MERFISH probe library and a poly(A) anchor probe. After hybridization, samples were embedded in a 4% polyacrylamide gel, optically cleared in a digestion buffer containing protease and mild detergent and stored at 4 °C until imaged.

MERFISH imaging of samples was performed on a homemade imaging platform. Before imaging, samples were stained with two segmentation markers, DAPI and an Alexa Fluor 488–conjugated readout probe complementary to the poly(A) anchor probe. For imaging, samples were held inside a flow chamber to accommodate buffer exchanges over the many rounds of MERFISH imaging. Each imaging round consisted of readout probe hybridization, imaging each FOV (220 µm × 220 µm per FOV) and readout probe fluorophore cleavage. Imaging consisted of 17 rounds. After imaging the segmentation markers in round 1, the barcode-encoded RNA species were imaged in rounds 2–12 (combinatorial smFISH rounds), and the individually labeled RNA species were imaged in rounds 13–16 (sequential smFISH rounds). In rounds 1–12, images of each FOV were acquired at seven focal planes separated by 1.5 µm in *z*. In rounds 13–16, images of each FOV were acquired at one focal plane 3.5 µm above the glass surface. In addition, every imaging round included a single *z*-plane image of the fiducial beads on the glass surface for image registration. The number of FOVs imaged for each sample varied based on the size of the sample.

Subsequently, all MERFISH image analysis was performed using the MERlin Python package (https://github.com/ZhuangLab/MERlin). First, for each FOV, the images from each imaging round were aligned to correct for *x*–*y* drift in the stage position. For the combinatorial rounds, image stacks for each FOV were high-pass filtered, deconvolved using Lucy–Richardson deconvolution and, finally, low-pass filtered. Individual RNA molecules were then identified by a pixel-based decoding method as previously described^11^. All cell segmentation was performed using the cellpose Python package (https://github.com/MouseLand/cellpose) using the ‘nuclei’ model applied to the DAPI image for each FOV. Identified individual RNA molecules were then assigned to individual cells based on if they were located within the segmented boundaries. For the sequential smFISH rounds, images were high-pass filtered and background subtracted, and the expression of each gene in each cell was calculated as the sum of the fluorescence intensity of all pixels within the segmentation boundary of the central *z*-plane of each cell. The signals from the eight sequential genes were merged with the RNA counts matrix from the 291 genes measured in the combinatorial smFISH rounds to generate a final expression matrix for each tissue slice. Each slice was then evaluated against QC criteria to determine if it would be included in further analysis. The QC criteria for each slice consisted of (1) the average number of RNA counts per cell (≥50 to pass) and (2) the Pearson correlation of the average gene expression between the MERFISH dataset and an scRNA-seq dataset derived from the same tumor (Pearson correlation coefficient ≥ 0.60 to pass). Both criteria had to be met to pass QC.

### Targeted ExSeq data generation

The detailed protocols for targeted ExSeq data generation are available as a protocols collection on https://www.protocols.io/ (ref. ^86^). The overall structure of the work is in three parts: experimental design, experimental execution and analysis. In the experimental design step, padlock probes were designed that targeted the genes identified above. In the experimental execution steps, tissue sections were fixed and expanded, followed by targeted in situ sequencing library preparation and in situ sequencing of the prepared library. Finally, in situ sequencing data were decoded to identify specific RNA transcripts in the specimen.

Padlock probes were designed that targeted the genes identified above, following the ‘Targeted ExSeq–Probe Generation’ protocol. In brief, logical barcode sequences of length 7, with each position in the barcode being a number between 0 and 3, were generated and randomly assigned to the genes of interest. These barcodes were designed to have a minimum Hamming distance of 3, enabling error detection and correction. These logical barcodes were then implemented as nucleic acid sequences on the backbone of the padlock probe, with one sequence for readout with the Illumina sequencing-by-synthesis chemistry (used in this work) and another sequence for readout with the SOLiD sequencing-by-ligation chemistry (not used here). Both sequences are included in the backbone of the probe adjacent to the sequencing primer site. Probe homology sequences were then generated by performing a sliding window search along each transcript. Candidate regions were excluded for sequence complexity (more than five consecutive repeated bases, containing three or fewer unique nucleotides, GC content outside of 40–65%), physical considerations (melting temperature (T_m_) of either arm of the padlock probe below a gene-specific T_m_ threshold, T_m_ difference between the two arms exceeding 8 °C, presence of hairpins or dimers in the homology region) or significant homology to a different transcript that spans the ligation junction. For each gene, the first 16 homology regions starting from the 5′ end of the transcript were selected. If fewer than 16 homology regions were identified, all were selected for use. Probes for each gene were assembled by combining the homology regions with a backbone sequence shared across all probes for that gene (containing the barcodes). Designed padlock probes were then purchased in plate-based format from IDT and pooled together.

The first experimental step was tissue preparation following the ‘Targeted ExSeq–Tissue Preparation’ protocol, following path C in the flowchart in the protocol abstract. In this step, tissue sections were fixed, expanded and prepared for targeted ExSeq library preparation. In brief, after cryosectioning onto Superfrost Plus glass slides (described above), tissue sections were fixed with ice-cold 10% formalin for 12 min and then washed three times for 5 min each wash with ice-cold 1× PBS. Slides were then stored in 70% ethanol and stored at 4 °C for up to 1 week. To begin gel embedding, slides were briefly dried with a laboratory wipe, and a Bio-Rad Frame-Seal sticker was placed around the tissue section, forming a chamber for washes. The tissue was rehydrated by washing with 1× PBS and then treated with 0.1 mg ml^−1^ LabelX overnight at 37 °C to enable nucleic acid anchoring into the expansion hydrogel. The tissue was then embedded into the expansion microscopy hydrogel and digested following the Robust Digestion Conditions described in the protocol. After digestion, the sample was expanded and re-embedded into a non-expanding polyacrylamide gel to lock in the expansion factor. The fixed charge of the carboxylates in the original expansion gel was then chemically passivated using EDC-NHS activation of carboxylate groups, followed by amide bond formation with ethanolamine. Gels were then trimmed to size.

The second experimental step was library preparation following the ‘Targeted ExSeq–Sequencing Library Preparation’ protocol. In brief, padlock probes bearing barcode sequences are hybridized to RNA transcripts. Padlock probes are then enzymatically circularized using SplintR Ligase and then enzymatically amplified using rolling circle amplification using Phi29 DNA Polymerase, forming amplicons (also called RCA colonies, or rolonies). The amplicons are then cross-linked to each other and the sample and are ready for in situ sequencing. For these samples, the universal amplicon detection hybridization step was skipped here and performed after in situ sequencing was completed.

The third experimental step was in situ sequencing following the ‘Targeted ExSeq–In Situ Sequencing (Illumina Chemistry)’ protocol. In brief, samples (gel-embedded tissues with in situ sequencing libraries) were covalently anchored to glass-bottom plates for imaging by functionalizing the plate surface with acryloyl groups, placing the specimen gel inside the well and casting a second re-embedding gel that anchored the specimen gel to the glass-bottom plate. The sample was then prepared for sequencing by capping free 3′ ends of DNA in the sample with dideoxy nucleotides using TdT tailing. The Illumina sequencing primer was hybridized to amplicons within the specimen, and seven rounds of Illumina sequencing-by-synthesis were performed in situ using reagents collected from MiSeq version 3 sequencing kits. Each round of sequencing consisted of base incorporation (addition of the next base), four-color imaging of the amplicons on a spinning disk confocal microscope and cleavage of the reversible terminator, enabling the next round of sequencing to be performed. After the final round of sequencing, the universal amplicon detection probe was hybridized to the sample (see library preparation protocol), and a final round of imaging was performed.

Data analysis to convert in situ sequencing images to localized reads in space was performed using the established ExSeqProcessing pipeline (https://github.com/dgoodwin208/ExSeqProcessing) using the Big Experiment (BigEXP) approach for image registration after color correction and normalization. After image registration is puncta extraction and base calling, using the probe barcodes as the reference library. Manual cell segmentation was performed in 2D by using VASTLite version 1.3.0 (ref. ^87^) to manually annotate nuclei boundaries of a 2D maximum intensity projection image of the DAPI channel. Reads localized within nuclei were assigned to that cell; reads outside of segmented nuclei were discarded. The quality of each sample was evaluated, and samples with an average read count per cell lower than 50 were excluded from further analysis.

### Processing and quality assurance of the spatial expression data

All spatial expression data were received in their respective typical formats. In a first step, all data types were transferred into a common observation × feature matrix format following the format of scRNA-seq data. For single-molecule data (MERFISH and ExSeq), two matrices were created, one cell × feature matrix using the accompanying cell segmentation information and one bin × feature matrix where expression was represented per 10 μm × 10-μm bin, resembling Slide-seq data. Additionally, spatial coordinates were adjusted to all start at [0 | 0] and scaled to represent a positional resolution of 1 pixel per μm, which was the lowest original resolution of the data. Note that, in spatial expression data, we distinguish between ‘positional resolution’ and ‘capture resolution’: positional resolution is the resolution at which the position in space of an observation or molecule is reported, whereas the capture resolution is the resolution at which molecules are distinctly captured. For example, in Slide-seq, the positional resolution (that is, the resolution at which the position of the beads is reported) is 0.65 μm per pixel, and the capture resolution is 10 μm (=diameter of a bead) because molecules that get captured by the same bead have a maximum distance of 10 μm from each other. For single-molecule resolved methods, positional and capture resolution are identical.

Having brought all data into the same format allowed their efficient processing together with the matching sc/snRNA-seq data as an anndata object using SCANPY^88^. This way, for each patient and method, one anndata object was created and processed individually. The same measures were applied on all data types as reasonable given the differences in design parameters between the different methods.

Quality filtering was applied using the SCANPY version 1.7.2 functions filter_cells with method-specific parameters and filter_genes with the min_cells parameter set to 3. The following filter_cells parameters were used: min_counts = 20 and min_genes = 1 (MERFISH and ExSeq), min_counts = 30 and min_genes = 30 (initial Slide-seq and sc/snRNA-seq). For Slide-seq and sc/snRNA-seq, an additional iterative process of step-wise min_counts parameter increase was performed to ensure that the fraction of low-quality beads with fewer than 100 counts retained in the data did not surpass 35%. This adaptive procedure ensured sufficient quality while retaining the maximum number of observations possible. This procedure was also performed on sc/snRNA-seq data that had already been quality filtered as described above to ensure equivalent filtering in the extremely unlikely case that this procedure might prove to be more stringent in specific cases. For CODEX, the parameter settings min_counts = 1 and min_genes = 1 were used, translating into a requirement of a value of greater than 1 in at least one gene, essentially disabling this filtering step for these intensity-based data, because cell quality filtering had already taken place during the segmentation process.

After filtering, the SCANPY workflow, including normalization, log1p transformation, scaling, highly variable gene selection, regression of total counts and mitochondrial counts (where possible), PCA, nearest neighbor finding, Leiden clustering and 2D projection using UMAP, was applied. For CODEX, normalization and regression were not performed given the intensity-based (not count-based) nature of the data and the within-sample scope of this analysis.

Finally, the spatial expression data and H&E images were aligned in a semi-manual process to honor their serial nature and allow efficient comparison as well as transfer of histopathological annotations from the H&E images. To this end, we devised custom functions that allow for all necessary transformations (rotation, translocation, flipping and scaling) and, using Jupyter notebooks, manually found and recorded the respective parameters for each sample until all data from one biopsy were adequately registered to a common coordinate system in a reproducible manner. To filter out spurious measurements, all observations that resided outside of the area covered by the H&E section were removed, and observations were annotated according to the histopathological annotation that they overlapped.

### Cell type annotation of the spatial expression data by annotation transfer from the sc/snRNA-seq data

For all spatial expression data, cell types were annotated using the TACCO framework version 0.0.1 (ref. ^44^) together with the matching sc/snRNA-seq data as reference. Specifically, we used two conceptually different annotation methods wrapped in the TACCO framework that are both able to deconvolve cell type mixtures. We used RCTD version 1.2.0 (ref. ^43^) as a previously published, well-accepted tool that was designed for the annotation of Slide-seq data and that explicitly models cell-type-specific read count distributions to determine the cell type composition of observations. We also used TACCOʼs own annotation method, which is based on unbalanced optimal transport (OT), which makes fewer assumptions about the properties of the input data and, in particular, is not, per design, limited to count data, which is necessary for a coherent annotation, including the CODEX data. RCTD was run with default parameters except for min_ct = 2. OT was run with lamb = 0.001 and ‘boosted’ by using TACCO’s platform normalization, multicenter (multi_center=4 ) and bisectioning (bisections = 4, bisection_divisor = 3) functionalities. Per observation, compositional as well as categorical (maximum cell type) annotations were stored for further use.

### Cell type frequency correlation analysis

To assess the agreement of local cell type frequencies across the serial sections of the same biopsies profiled with different methods, we defined, for each biopsy, a universal grid of 100 × 100-μm bins, and, within each bin and section, the cell type composition was calculated based on the previously assigned categorical cell type annotations, yielding, for each bin and section, a vector of cell type frequencies with the length of the cell types seen in any of the sections of a given biopsy. Pair-wise Pearson correlations were then calculated per bin between the cell type composition vectors derived from each of the sections, representing different profiling methods and/or replicates.

### Analysis of cluster congruence using the ARI

To assess congruence of expression-based Leiden clusters and cell type or patient/sample annotations, respectively, with the assessed communities (Leiden clusters, patients/samples and cell types) consisting of individual observations (single cells/beads/bins), the ARI was calculated using the function adjusted_rand_score from the Python package scikit-learn version 0.24.1. Bootstrapping across 10 iterations was used for statistical robustness, and results are reported as mean and standard deviation.

### Cell type co-localization analysis

Cell type co-localization analysis was performed using TACCO’s version 0.2.2 co_occurrence function based on the compositional OT annotations for a distance of up to 500 μm and using the ‘log_occ’ score. In brief, at each distance from a selected central cell type (here, macrophages), the function calculates the probability of finding the other annotated cell types relative to the case where a central cell type is not selected. Two scores were then derived from the co-localization score: co-localization strength, defined as the score of the first distance interval, and co-localization range, defined as the score at the distance interval where the score has decayed to 25% of the score in the first distance interval.

### De novo cell type annotation of the cell-segmented MERFISH data

Leveraging the single-cell-like behavior of the cell-segmented MERFISH data, in addition to the annotation transfer as described above, we performed manual cluster-wise and marker gene-based annotation as is frequently done in scRNA-seq data. To this end, all cell-segmented MERFISH data were combined into one anndata object and processed using SCANPY version 1.7.2 functions as described above. For consistency, we used a similar level of resolution for the annotated cell types as was used in the sc/snRNA-seq annotation and assigned new cell type labels only when clusters clearly displayed features that did not match to any previously annotated cell types, which was the case for a small population of potentially regulatory B cells expressing FOXP3 in addition to the typical B cell marker FCRL5.

### Characterization of macrophage subclusters

To characterize macrophage subclusters in each profiling method, Leiden clusters were called on Harmony-aligned data (as described in the subsection ‘Integration of sc/snRNA-seq data or spatial data on a pseudobulk or single-cell/bead/bin level’). Differentially expressed genes were called using the function rank_genes_groups of the Python package SCANPY version 1.7.2 with the method parameter set to ‘wilcoxon’ and default parameters otherwise. One or two of the top five differentially expressed genes were selected for display.

### Differential expression analysis between EMT phenotypes

To detect differentially expressed genes among the three spatial phenotypes (EMT-high, EMT-low and EMT-patched), the function ‘enrichments’ of the Python package TACCO version 0.2.2 was used in a one-against-all-others or EMT-high versus EMT-patched setup with the following relevant parameters: p_corr = ‘fdr_bh’ (multiple testing correction using Benjamini–Hochberg correction), position_split = (1,2) (split sample in two parts along the *y* axis to capture within-sample variability), method = ‘welch’ (Welch’s *t*-test for statistical significance testing), direction = ‘both’ (test for increased/enriched or decreased/depleted expression), reduction = ‘mean’ (measure to calculate pseudobulk values across sample splits) and normalization = ‘clr’ (use center log-ratio normalization).

### Differential cell type composition analysis between EMT defined neighborhoods

To detect differences in cell type composition between EMT-high and EMT-low neighborhoods, a two-sided Wilcoxon test and Benjamini–Hochberg multiple testing correction were applied on center log-ratio normalized, cell type compositions in 100 × 100-μm bins. EMT-high and EMT-low neighborhoods were defined as 100 × 100-μm bins with a mean EMT score greater (high) or smaller (low) than the median EMT score for a given sample.

### MERFISH-based differential expression analysis between T/NK proximal and distal malignant cells

To investigate differences in expression profiles of malignant cells that are located in proximity of T or NK cells and those that are not, we used the cell-segmented and manually annotated MERFISH data and defined T/NK high-malignant cells as those that reside in a 100 × 100-μm bin together with at least one T or NK cell and the T/NK low-malignant cells as those that reside in a 100 × 100-μm bin that does not contain a T or NK cells. We then ran the SCANPY version 1.7.2 function rank_genes_groups using the Wilcoxon test and Benjamini–Hochberg correction to compare both groups of malignant cells and rank the genes by their expression difference. This analysis was performed in a sample-specific setup as well as a combined setup across all samples.

### Statistical analysis

Box plots follow the standard format (center line corresponds to the median; box limits correspond to the upper and lower quartiles; whiskers represent the 1.5× interquartile range; points represent outliers). Where there were too many data points to show individually, width-scaled violin plots were used to represent the distribution of data points, where graphically possible (otherwise only box plots are shown).

Pearson correlation and Spearman correlation coefficients were calculated using the cor or cor.test function from the R package ‘stats’ or the corr function of the Python package ‘pandas’ version 1.1.3.

All UMAPs were created using SCANPY’s version 1.7.2 umap function with default parameters.

### Reporting summary

Further information on research design is available in the Nature Portfolio Reporting Summary linked to this article.

## Online content

Any methods, additional references, Nature Portfolio reporting summaries, source data, extended data, supplementary information, acknowledgements, peer review information; details of author contributions and competing interests; and statements of data and code availability are available at 10.1038/s41591-024-03215-z.

## Supplementary information

Supplementary Figs. 1–5.

Reporting Summary

Supplementary Tables 1–5.

## Data Availability

All data can be retrieved from Synapse or the database of Genotypes and Phenotypes (dbGaP) (accession number: phs002371) through the HTAN Portal at https://humantumoratlas.org and the associated HTAN Publication Page https://humantumoratlas.org/publications/htapp_mbc_klughammer_2024. For convenience, processed data are additionally available from the Single-Cell Portal (https://singlecell.broadinstitute.org/single_cell/study/SCP2702) and interactively browsable through CELLxGENE (https://cellxgene.cziscience.com/collections/a96133de-e951-4e2d-ace6-59db8b3bfb1d). The pre-built Cell Ranger reference GRCh38 version 3.0.0 (November 2016) in its spliced (scRNA-seq) and pre-mRNA (snRNA-seq) version was provided by 10x Genomics (https://www.10xgenomics.com/support/software/cell-ranger/latest/release-notes/cr-reference-release-notes).

## Code availability

Code used to perform the presented analysis is available on GitHub: https://github.com/klarman-cell-observatory/HTAPP-Pipelines/tree/master/HTAPP_MBC.
