# Author Correction: A multi-modal single-cell and spatial expression map of metastatic breast cancer biopsies across clinicopathological features

**DOI:** 10.1038/s41591-025-03665-z

**Published:** 2025-03-28

**Authors:** Johanna Klughammer, Daniel L. Abravanel, Åsa Segerstolpe, Timothy R. Blosser, Yury Goltsev, Yi Cui, Daniel R. Goodwin, Anubhav Sinha, Orr Ashenberg, Michal Slyper, Sébastien Vigneau, Judit Jané‐Valbuena, Shahar Alon, Chiara Caraccio, Judy Chen, Ofir Cohen, Nicole Cullen, Laura K. DelloStritto, Danielle Dionne, Janet Files, Allison Frangieh, Karla Helvie, Melissa E. Hughes, Stephanie Inga, Abhay Kanodia, Ana Lako, Colin MacKichan, Simon Mages, Noa Moriel, Evan Murray, Sara Napolitano, Kyleen Nguyen, Mor Nitzan, Rebecca Ortiz, Miraj Patel, Kathleen L. Pfaff, Caroline B. M. Porter, Asaf Rotem, Sarah Strauss, Robert Strasser, Aaron R. Thorner, Madison Turner, Isaac Wakiro, Julia Waldman, Jingyi Wu, Jorge Gómez Tejeda Zañudo, Diane Zhang, Nancy U. Lin, Sara M. Tolaney, Eric P. Winer, Edward S. Boyden, Fei Chen, Garry P. Nolan, Scott J. Rodig, Xiaowei Zhuang, Orit Rozenblatt-Rosen, Bruce E. Johnson, Aviv Regev, Nikhil Wagle

**Affiliations:** 1 Klarman Cell Observatory, Broad Institute of Harvard and MIT, Cambridge, MA USA; 2 Department of Medical Oncology, Dana-Farber Cancer Institute, Boston, MA USA; 3 Harvard Medical School, Boston, MA USA; 4 Department of Chemistry and Chemical Biology, Harvard University, Cambridge, MA USA; 5 Baxter Laboratory in Stem Cell Biology, Department of Microbiology and Immunology, Stanford University School of Medicine, Stanford, CA USA; 6 Department of Media Arts and Sciences, McGovern Institute, Massachusetts Institute of Technology, Cambridge, MA USA; 7 Center for Cancer Genomics, Dana-Farber Cancer Institute, Boston, MA USA; 8 Faculty of Engineering, Gonda Brain Research Center and Institute of Nanotechnology, Bar-Ilan University, Ramat Gan, Israel; 9 Broad Institute of Harvard and MIT, Cambridge, MA USA; 10 Center for Immuno-Oncology, Dana-Farber Cancer Institute, Boston, MA USA; 11 School of Computer Science and Engineering, The Hebrew University of Jerusalem, Jerusalem, Israel; 12 Racah Institute of Physics, The Hebrew University of Jerusalem, Jerusalem, Israel; 13 Faculty of Medicine, The Hebrew University of Jerusalem, Jerusalem, Israel; 14 Department of Media Arts and Sciences, Massachusetts Institute of Technology, Cambridge, MA USA; 15 Department of Biological Engineering, Massachusetts Institute of Technology, Cambridge, MA USA; 16 Department of Biology, Koch Institute for Integrative Cancer Research, Massachusetts Institute of Technology, Cambridge, MA USA; 17 Howard Hughes Medical Institute, Chevy Chase, MD USA; 18 Department of Brain and Cognitive Sciences, Massachusetts Institute of Technology, Cambridge, MA USA; 19 K. Lisa Yang Center for Bionics, Massachusetts Institute of Technology, Cambridge, MA USA; 20 Department of Stem Cell and Regenerative Biology, Harvard University, Cambridge, MA USA; 21 Department of Pathology, Brigham and Women’s Hospital, Boston, MA USA; 22 Department of Pathology, Dana-Farber Cancer Institute, Boston, MA USA; 23 Department of Physics, Harvard University, Cambridge, MA USA; 24 Present Address: Gene Center and Department of Biochemistry, Ludwig Maximilians Universität München, Munich, Germany; 25 Present Address: Department of Microbiology, Immunology and Genetics, Faculty of Health Sciences, Ben-Guiron University, Beersheba, Israel; 26 Present Address: AstraZeneca R&D, Boston, MA USA; 27 Present Address: Department of Microbiology, Immunology, and Cancer Biology, University of Virginia, Charlottesville, VA USA; 28 Present Address: Genentech, Inc., South San Francisco, CA USA

**Keywords:** Breast cancer, Cancer genomics, Data integration, Transcriptomics

Correction to: *Nature Medicine* 10.1038/s41591-024-03215-z, published online 30 October 2024.

In the version of the article initially published, the text “We gratefully acknowledge LMU Klinikum for providing computing resources on their Clinical Open Research Engine (CORE), the Bioinformatic Core Facility of the Biomedical Center Munich for providing computing resources on their HPC system, and the German Research Foundation (DFG) funded CRC237 and CRC274 for additional support” was missing from the Acknowledgements section and has now been added to the HTML and PDF versions of the article.

