# Supplementary material for: A multi-modal single-cell and spatial expression map of metastatic breast cancer biopsies across clinicopathological features
Source: Nat Med. 2024 Oct 30;30(11):3236–49. doi: 10.1038/s41591-024-03215-z (PMC11564109; doi:10.1038/s41591-024-03215-z)
Supplement: Supplementary file 1 — Supplementary Figs. 1–5. [file 41591_2024_3215_MOESM1_ESM.pdf]

nature medicine

Supplementary information https://doi.org/10.1038/s41591-024-03215-z

A multi-modal single-cell and spatial expression map of metastatic breast cancer biopsies across clinicopathological features

In the format provided by the authors and unedited

Supplementary Information

Tables

Supplementary Table 1: Cell-ranger overview statistics for single-cell and single-nucleus RNAseq data.

Supplementary Table 2: Overview statistics and clinical annotations for all samples.

Supplementary Table 3: Annotation for the genes included in the MERFISH and ExSeq gene panel.

Supplementary Table 4: Variance of gene expression in malignant cells explained by technical and clinicopathological variables.

Supplementary Table 5: COXEX antibody panel.

Figures

Supplementary Figures 1-5: Detailed view of each biopsy with sc/snRNA-Seq, Slide-seq, MERFISH, ExSeq and CODEX

a) Spatial scatter plots for all spatial methods and available replicates, where each observation (cell, bead, bin) is colored by its TACCO-OT or RCTD annotated cell type, as well as the matched H&E image.

b) Barplots depicting cell type composition based on TACCO-OT or RCTD transferred annotations for all spatial methods and available replicates as well as the matching snRNA-Seq data as reference (cell_type).

c) Heatmaps depicting the degree of co-occurrence between all pairs of cell types within a radius of 50 μm based on TACCO-OT cell type annotations.

d) UMAPs of all data types based on their expression profiles, with observations colored by the original cell type annotation (snRNA-Seq) or TACCO-OT transferred cell type annotation (spatial methods).

S1: Biopsy 313-932
S2: Biopsy 514-6760
S3: Biopsy 880-7179
S4: Biopsy 917-4531
S5: Biopsy 982-7629

313-932

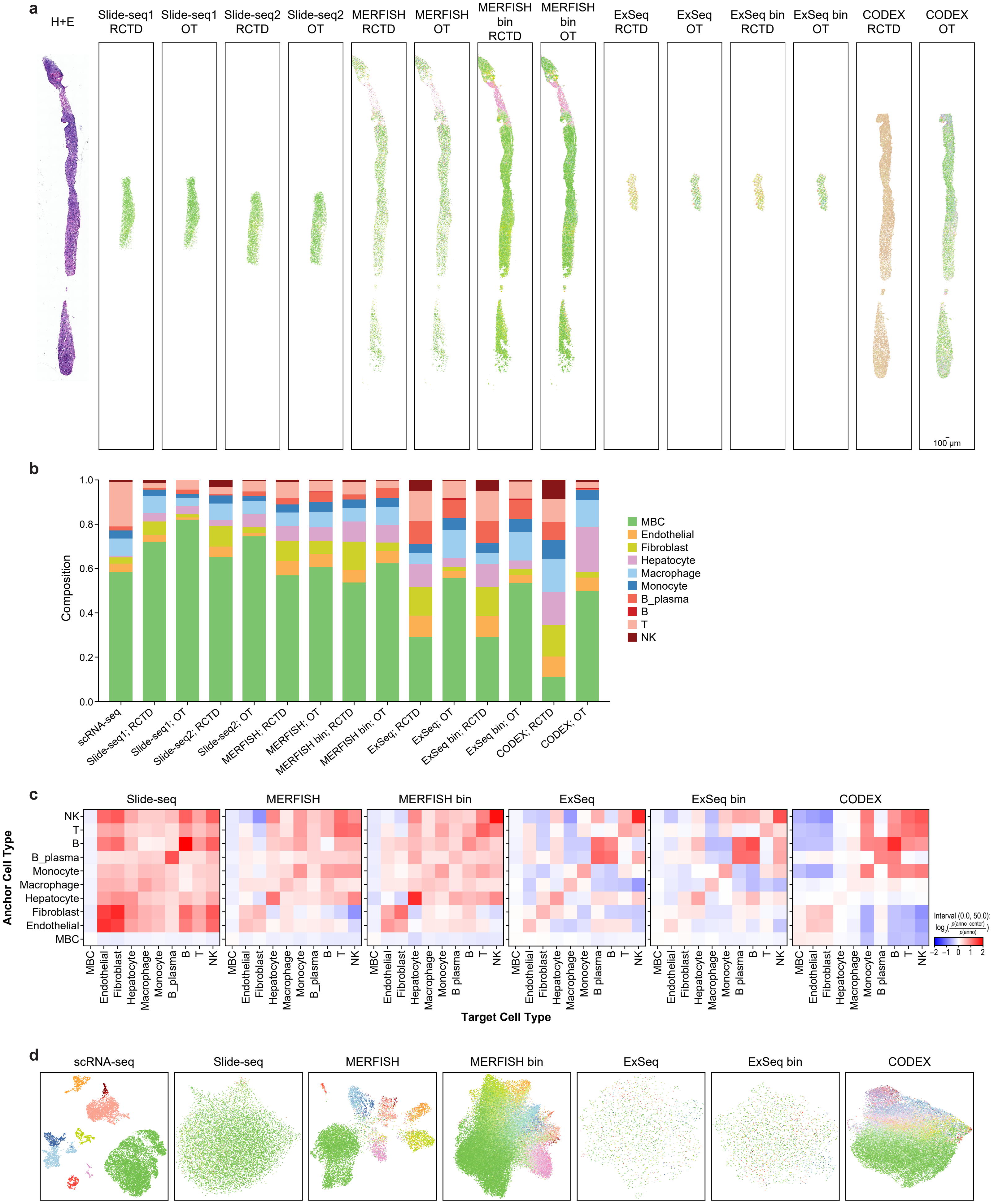
a
H+E
Slide-seq1 RCTD
Slide-seq1 OT
Slide-seq2 RCTD
Slide-seq2 OT
MERFISH RCTD
MERFISH OT
MERFISH bin RCTD
MERFISH bin OT
ExSeq RCTD
ExSeq OT
ExSeq bin RCTD
ExSeq bin OT
CODEX RCTD
CODEX OT
100 μm
b
Composition
MBC
Endothelial
Fibroblast
Hepatocyte
Macrophage
Monocyte
B_plasma
B
T
NK
scRNA-seq
Slide-seq1; RCTD
Slide-seq1; OT
Slide-seq2; RCTD
Slide-seq2; OT
MERFISH; RCTD
MERFISH; OT
MERFISH bin; RCTD
MERFISH bin; OT
ExSeq; RCTD
ExSeq; OT
ExSeq bin; RCTD
ExSeq bin; OT
CODEX; RCTD
CODEX; OT
c
Slide-seq
MERFISH
MERFISH bin
ExSeq
ExSeq bin
CODEX
Anchor Cell Type
Target Cell Type
Interval (0.0, 50.0):
d
scRNA-seq
Slide-seq
MERFISH
MERFISH bin
ExSeq
ExSeq bin
CODEX

514-6760

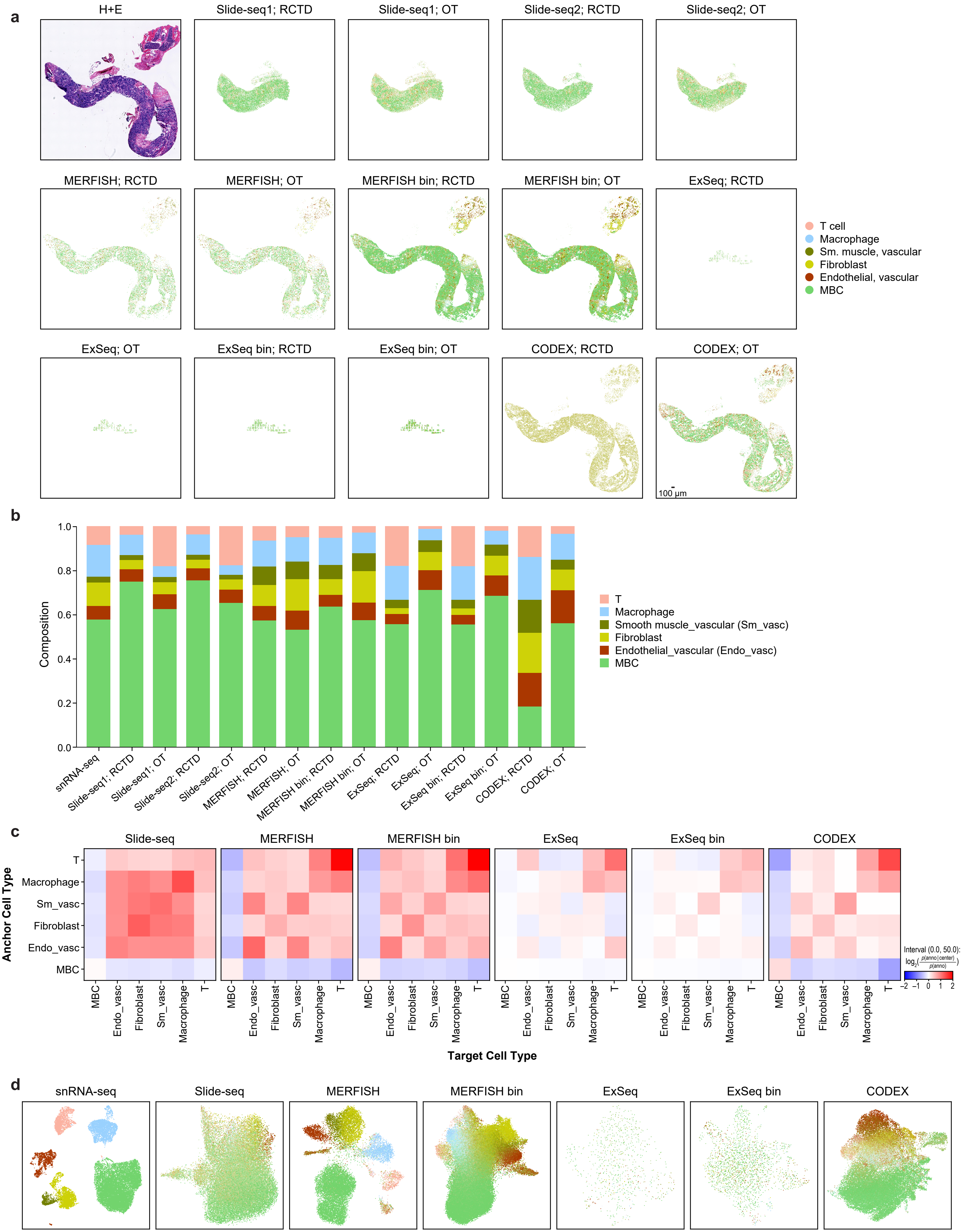
a
H+E
Slide-seq1; RCTD
Slide-seq1; OT
Slide-seq2; RCTD
Slide-seq2; OT
MERFISH; RCTD
MERFISH; OT
MERFISH bin; RCTD
MERFISH bin; OT
ExSeq; RCTD
T cell
Macrophage
Sm. muscle, vascular
Fibroblast
Endothelial, vascular
MBC
ExSeq; OT
ExSeq bin; RCTD
ExSeq bin; OT
CODEX; RCTD
CODEX; OT
100 µm
b
Composition
1.0
0.8
0.6
0.4
0.2
0.0
snRNA-seq
Slide-seq1; RCTD
Slide-seq1; OT
Slide-seq2; RCTD
Slide-seq2; OT
MERFISH; RCTD
MERFISH; OT
MERFISH bin; RCTD
MERFISH bin; OT
ExSeq; RCTD
ExSeq; OT
ExSeq bin; RCTD
ExSeq bin; OT
CODEX, RCTD
CODEX; OT
T
Macrophage
Smooth muscle_vascular (Sm_vasc)
Fibroblast
Endothelial_vascular (Endo_vasc)
MBC
c
Slide-seq
MERFISH
MERFISH bin
ExSeq
ExSeq bin
CODEX
Anchor Cell Type
T
Macrophage
Sm_vasc
Fibroblast
Endo_vasc
MBC
Target Cell Type
Interval (0.0, 50.0):
−2 −1 0 1 2
d
snRNA-seq
Slide-seq
MERFISH
MERFISH bin
ExSeq
ExSeq bin
CODEX

880-7179

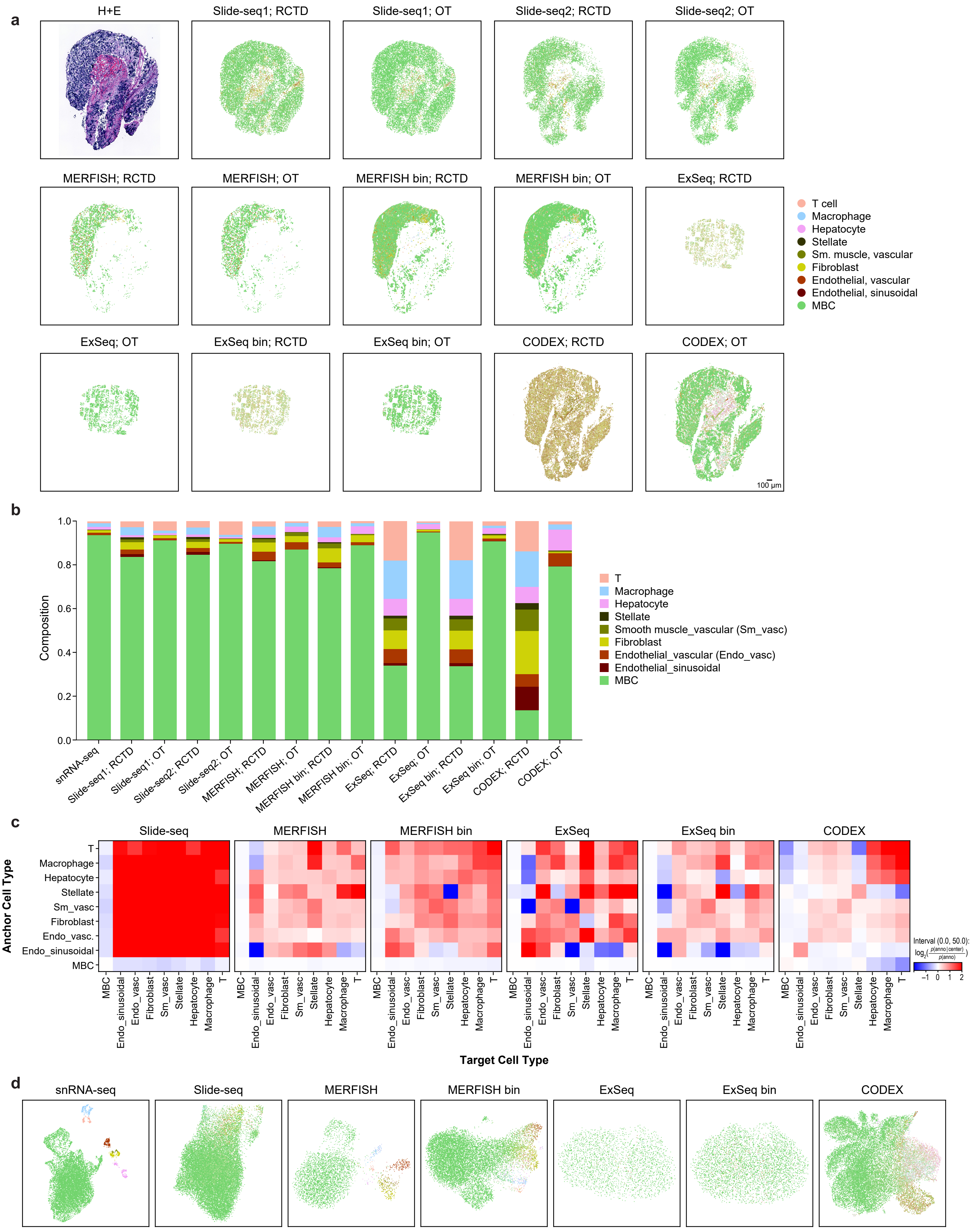

a
H+E
Slide-seq1; RCTD
Slide-seq1; OT
Slide-seq2; RCTD
Slide-seq2; OT
MERFISH; RCTD
MERFISH; OT
MERFISH bin; RCTD
MERFISH bin; OT
ExSeq; RCTD
ExSeq; OT
ExSeq bin; RCTD
ExSeq bin; OT
CODEX; RCTD
CODEX; OT
100 μm
T cell
Macrophage
Hepatocyte
Stellate
Sm. muscle, vascular
Fibroblast
Endothelial, vascular
Endothelial, sinusoidal
MBC
b
Composition
T
Macrophage
Hepatocyte
Stellate
Smooth muscle_vascular (Sm_vasc)
Fibroblast
Endothelial_vascular (Endo_vasc)
Endothelial_sinusoidal
MBC
snRNA-seq
Slide-seq1; RCTD
Slide-seq1; OT
Slide-seq2; RCTD
Slide-seq2; OT
MERFISH; RCTD
MERFISH; OT
MERFISH bin; RCTD
MERFISH bin; OT
ExSeq; RCTD
ExSeq; OT
ExSeq bin; RCTD
ExSeq bin; OT
CODEX; RCTD
CODEX; OT
c
Slide-seq
MERFISH
MERFISH bin
ExSeq
ExSeq bin
CODEX
Anchor Cell Type
T
Macrophage
Hepatocyte
Stellate
Sm_vasc
Fibroblast
Endo_vasc.
Endo_sinusoidal
MBC
Target Cell Type
MBC
Endo_sinusoidal
Endo_vasc
Fibroblast
Sm_vasc
Stellate
Hepatocyte
Macrophage
T
Interval (0.0, 50.0):
log2(p(anno|center)/p(anno))
d
snRNA-seq
Slide-seq
MERFISH
MERFISH bin
ExSeq
ExSeq bin
CODEX

917-4531

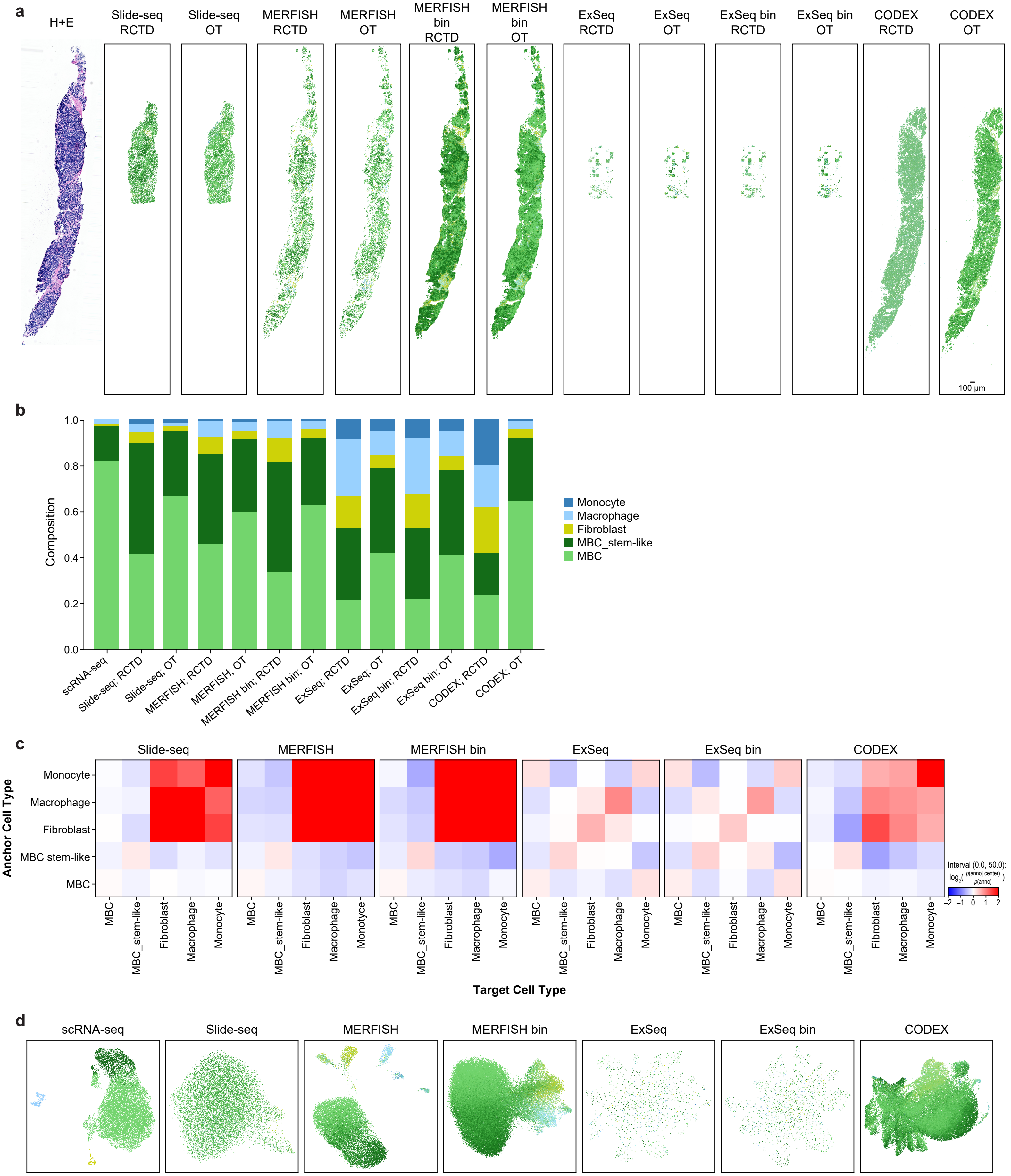

a
H+E
Slide-seq RCTD
Slide-seq OT
MERFISH RCTD
MERFISH OT
MERFISH bin RCTD
MERFISH bin OT
ExSeq RCTD
ExSeq OT
ExSeq bin RCTD
ExSeq bin OT
CODEX RCTD
CODEX OT
100 µm
b
Composition
1.0
0.8
0.6
0.4
0.2
0.0
scRNA-seq
Slide-seq, RCTD
Slide-seq, OT
MERFISH, RCTD
MERFISH, OT
MERFISH bin, RCTD
MERFISH bin, OT
ExSeq, RCTD
ExSeq, OT
ExSeq bin, RCTD
ExSeq bin, OT
CODEX, RCTD
CODEX, OT
Monocyte
Macrophage
Fibroblast
MBC_stem-like
MBC
c
Slide-seq
MERFISH
MERFISH bin
ExSeq
ExSeq bin
CODEX
Anchor Cell Type
Monocyte
Macrophage
Fibroblast
MBC stem-like
MBC
MBC
MBC_stem-like
Fibroblast
Macrophage
Monocyte
Monotyce
Target Cell Type
Interval (0.0, 50.0):
−2 −1 0 1 2
d
scRNA-seq
Slide-seq
MERFISH
MERFISH bin
ExSeq
ExSeq bin
CODEX

982-7629

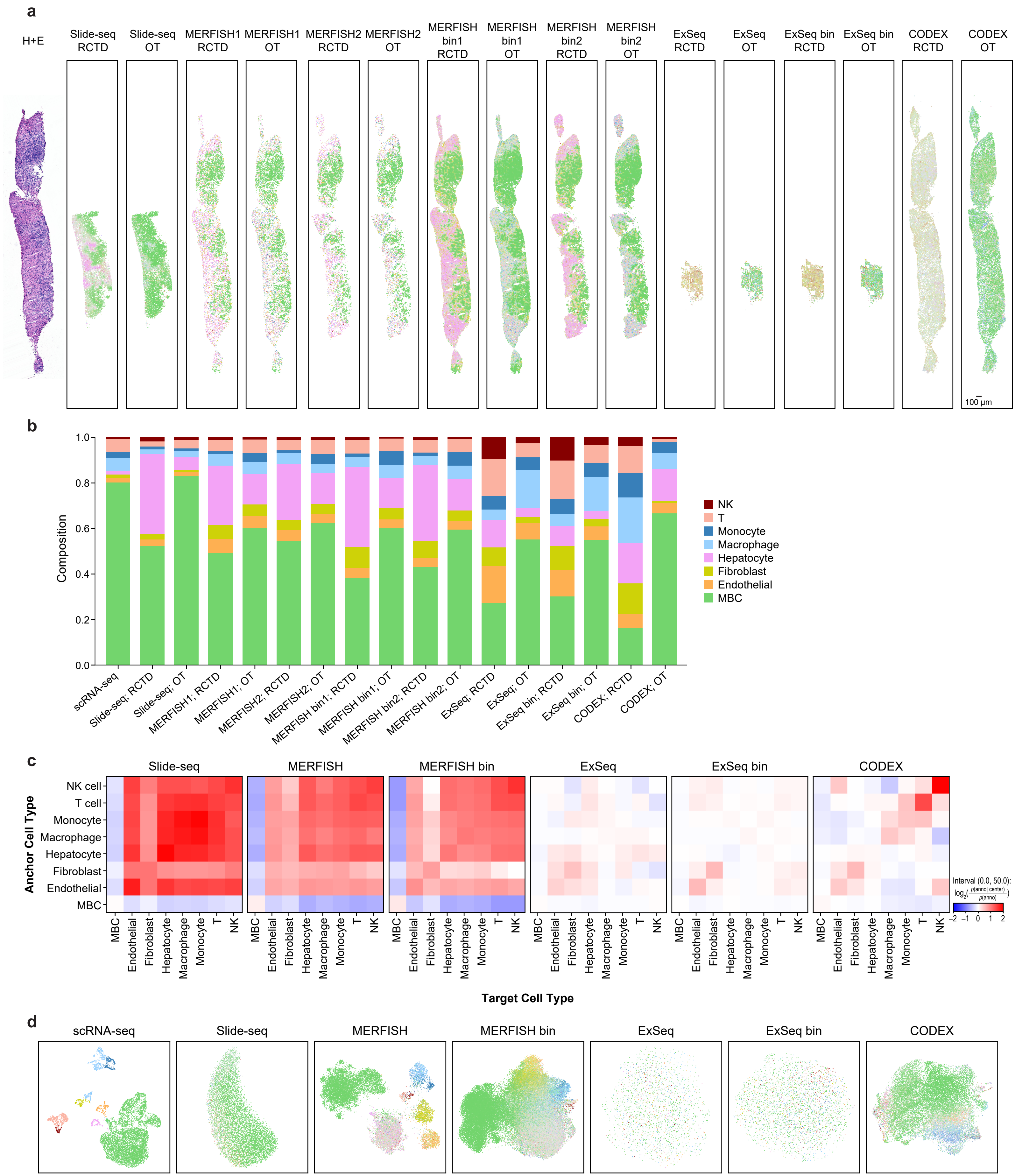

a
H+E
Slide-seq RCTD
Slide-seq OT
MERFISH1 RCTD
MERFISH1 OT
MERFISH2 RCTD
MERFISH2 OT
MERFISH bin1 RCTD
MERFISH bin1 OT
MERFISH bin2 RCTD
MERFISH bin2 OT
ExSeq RCTD
ExSeq OT
ExSeq bin RCTD
ExSeq bin OT
CODEX RCTD
CODEX OT
100 μm
b
Composition
1.0
0.8
0.6
0.4
0.2
0.0
scRNA-seq
Slide-seq; RCTD
Slide-seq; OT
MERFISH1; RCTD
MERFISH1; OT
MERFISH2; RCTD
MERFISH2; OT
MERFISH bin1; RCTD
MERFISH bin1; OT
MERFISH bin2; RCTD
MERFISH bin2; OT
ExSeq; RCTD
ExSeq; OT
ExSeq bin; RCTD
ExSeq bin; OT
CODEX; RCTD
CODEX; OT
NK
T
Monocyte
Macrophage
Hepatocyte
Fibroblast
Endothelial
MBC
c
Slide-seq
MERFISH
MERFISH bin
ExSeq
ExSeq bin
CODEX
Anchor Cell Type
NK cell
T cell
Monocyte
Macrophage
Hepatocyte
Fibroblast
Endothelial
MBC
Target Cell Type
MBC
Endothelial
Fibroblast
Hepatocyte
Macrophage
Monocyte
T
NK
Interval (0.0, 50.0):
log2(p(anno|center)/p(anno))
-2 -1 0 1 2
d
scRNA-seq
Slide-seq
MERFISH
MERFISH bin
ExSeq
ExSeq bin
CODEX
