# Supplementary material for: A multi-modal single-cell and spatial expression map of metastatic breast cancer biopsies across clinicopathological features
Source: Nat Med. 2024 Oct 30;30(11):3236–49. doi: 10.1038/s41591-024-03215-z (PMC11564109; doi:10.1038/s41591-024-03215-z)
Supplement: Supplementary file 2 — Reporting Summary [file 41591_2024_3215_MOESM2_ESM.pdf]

nature portfolio

Corresponding author(s): Johanna Klughammer, Daniel Abravanel, Aviv Regev and Nikhil Wagle

Last updated by author(s): 7/15/2024

Reporting Summary

Nature Portfolio wishes to improve the reproducibility of the work that we publish. This form provides structure for consistency and transparency in reporting. For further information on Nature Portfolio policies, see our Editorial Policies and the Editorial Policy Checklist.

Statistics

For all statistical analyses, confirm that the following items are present in the figure legend, table legend, main text, or Methods section.

n/a	Confirmed	
☐	☒	The exact sample size (*n*) for each experimental group/condition, given as a discrete number and unit of measurement
☐	☒	A statement on whether measurements were taken from distinct samples or whether the same sample was measured repeatedly
☐	☒	The statistical test(s) used AND whether they are one- or two-sided<br>*Only common tests should be described solely by name; describe more complex techniques in the Methods section.*
☐	☒	A description of all covariates tested
☐	☒	A description of any assumptions or corrections, such as tests of normality and adjustment for multiple comparisons
☐	☒	A full description of the statistical parameters including central tendency (e.g. means) or other basic estimates (e.g. regression coefficient) AND variation (e.g. standard deviation) or associated estimates of uncertainty (e.g. confidence intervals)
☐	☒	For null hypothesis testing, the test statistic (e.g. *F*, *t*, *r*) with confidence intervals, effect sizes, degrees of freedom and *P* value noted<br>*Give P values as exact values whenever suitable.*
☒	☐	For Bayesian analysis, information on the choice of priors and Markov chain Monte Carlo settings
☒	☐	For hierarchical and complex designs, identification of the appropriate level for tests and full reporting of outcomes
☐	☒	Estimates of effect sizes (e.g. Cohen's *d*, Pearson's *r*), indicating how they were calculated

Our web collection on statistics for biologists contains articles on many of the points above.

Software and code

Policy information about availability of computer code

Data collection	sc/snRNA-Seq: cellRanger 3.0.2<br>Slide-Seq: https://github.com/MacoskoLab/slideseq-tools<br>CODEX: https://github.com/nolanlab/CODEX, CellVisionSegmenter, ImageJ<br>MERFISH: https://github.com/ZhuangLab/MERlin 0.0.2.8, cellpose 0.1.7<br>ExSeq: https://github.com/dgoodwin208/ExSeqProcessing, VASTLite 1.3.0
Data analysis	R packages: SingleR 1.0.1, 1.0.3, inferCNV 1.2.0, LiblineaR 2.10-8, randomForest 4.6-14, variancePartition 1.14.0, CountClust 1.12.0, Enrichr 1.0, LIGER 0.5.0.9000, Seurat 2.3.4 (gene-panel design for MERSIFH and ExSeq), Seurat 3.1.1 (all other), RCTD 1.2.0, sva v3.34.0, genefu v2.20.0<br>Python packages: Scanpy 1.7.2, TACCO 0.0.1, 0.2.2, CellBender 0.1.0, scrublet 0.2.1, Harmony-pytorch v0.1.4, bbknn v1.5.1,scikit-learn v0.24.1, pandas v1.1.3<br>Custom code: https://github.com/klarman-cell-observatory/HTAPP-Pipelines/tree/master/HTAPP_MBC

For manuscripts utilizing custom algorithms or software that are central to the research but not yet described in published literature, software must be made available to editors and reviewers. We strongly encourage code deposition in a community repository (e.g. GitHub). See the Nature Portfolio guidelines for submitting code & software for further information.

Data

Policy information about availability of data

All manuscripts must include a data availability statement. This statement should provide the following information, where applicable:

- Accession codes, unique identifiers, or web links for publicly available datasets
- A description of any restrictions on data availability
- For clinical datasets or third party data, please ensure that the statement adheres to our policy

All data presented in this work can be browsed and downloaded through cellxgene:
https://cellxgene.cziscience.com/collections/a96133de-e951-4e2d-ace6-59db8b3bfb1d

Research involving human participants, their data, or biological material

Policy information about studies with human participants or human data. See also policy information about sex, gender (identity/presentation), and sexual orientation and race, ethnicity and racism.

Reporting on sex and gender	While both male and female patients were eligible to participate, the sex of all participants in the study was female, as expected based on the epidemiology of breast cancer. Gender was not considered during patient recruitment, sample selection or analysis.
Reporting on race, ethnicity, or other socially relevant groupings	Race, ethnicity and other social groupings were not considered during patient recruitment, sample selection or analysis.
Population characteristics	All patients on this study were adults ages 28-75 with metastatic breast cancer. Relevant clinical annotations including, for example, biopsy site, receptor status, time since metastatic diagnosis, and most recent treatment class can be found in Table S2.
Recruitment	All patients seen at Dana-Farber Cancer Institute for metastatic breast cancer who were scheduled to undergo a clinical tumor biopsy at the discretion of their treating physician at DFCI were eligible for this study, including all receptor subtypes and prior treatment histories in order to span the clinical and phenotypic heterogeneity of the disease. Eligible patients were approached regarding participation based on DF/HCC protocol 05-246. There were no additional systematic biases in recruitment/selection.
Ethics oversight	All samples included in this study were voluntarily donated by patients who provided informed consent under an IRB-approved protocol (DF/HCC #05-246) which includes permission for sample acquisition, clinical data abstraction, sample analysis, and data sharing. Analysis of biospecimens at the Broad Institute was performed under Broad Institute protocol #15-370B.

Note that full information on the approval of the study protocol must also be provided in the manuscript.

Field-specific reporting

Please select the one below that is the best fit for your research. If you are not sure, read the appropriate sections before making your selection.

☒ Life sciences ☐ Behavioural & social sciences ☐ Ecological, evolutionary & environmental sciences

For a reference copy of the document with all sections, see nature.com/documents/nr-reporting-summary-flat.pdf

Life sciences study design

All studies must disclose on these points even when the disclosure is negative.

Sample size	All high-quality samples available in the data collection period from 2018-2019 were included. This study is intended as pilot study to cover the clinical and histophathological diversity of MBC. Representations of some specific characteristics were therefor small. Analyses were chosen accordingly.
Data exclusions	During quality filtering, low quality cells (insufficient number of genes, inconclusive expression signals) were removed as described in the methods section.<br>sc/snRNAseq: Samples with extremely low numbers of cells or genes were excluded.<br>Slide-seq: The quality of all samples was evaluated and samples with an average read count per bead lower than 150 as well as those with an unrecognizable shape (which prevented spatial alignment) were excluded from further analysis.<br>CODEX:Finally, all samples were checked for unexpected signal appearance and distribution, but all samples were deemed inconspicuous and thus included in further analysis.<br>MERFISH:The QC criteria for each slice consisted of: 1) the average number of RNA counts per cell (>= 50 to pass) and 2) the Pearson correlation of the average gene expression between the MERFISH dataset and a scRNAseq dataset derived from the same tumor (Pearson correlation coefficient >= 0.60 to pass). Both criteria had to be met to pass QC.

	ExSeq:The quality of all samples was evaluated and samples with an average read count per cell lower than 50 were excluded from further analysis.
Replication	For sc/snRNAseq, replicates from each tumor biopsy were not feasible given the limited amount of tissue. For spatial expression profiling, multiple tissue sections were profiled by the same assay when possible (n=1-3 sections). All technically successful replicates are included in the dataset; some replicates were excluded due to insufficient data quality (see above and Supplementary Figures 1-5).
Randomization	No experimental groups were assigned in this study.
Blinding	No experimental groups were assigned in this study.

Reporting for specific materials, systems and methods

We require information from authors about some types of materials, experimental systems and methods used in many studies. Here, indicate whether each material, system or method listed is relevant to your study. If you are not sure if a list item applies to your research, read the appropriate section before selecting a response.

Materials & experimental systems

n/a	Involved in the study	
☒	☐	Antibodies
☒	☐	Eukaryotic cell lines
☒	☐	Palaeontology and archaeology
☒	☐	Animals and other organisms
☒	☐	Clinical data
☒	☐	Dual use research of concern
☒	☐	Plants

Methods

n/a	Involved in the study	
☒	☐	ChIP-seq
☒	☐	Flow cytometry
☒	☐	MRI-based neuroimaging

Plants

Seed stocks	*Report on the source of all seed stocks or other plant material used. If applicable, state the seed stock centre and catalogue number. If plant specimens were collected from the field, describe the collection location, date and sampling procedures.*
Novel plant genotypes	*Describe the methods by which all novel plant genotypes were produced. This includes those generated by transgenic approaches, gene editing, chemical/radiation-based mutagenesis and hybridization. For transgenic lines, describe the transformation method, the number of independent lines analyzed and the generation upon which experiments were performed. For gene-edited lines, describe the editor used, the endogenous sequence targeted for editing, the targeting guide RNA sequence (if applicable) and how the editor was applied.*
Authentication	*Describe any authentication procedures for each seed stock used or novel genotype generated. Describe any experiments used to assess the effect of a mutation and, where applicable, how potential secondary effects (e.g. second site T-DNA insertions, mosiacism, off-target gene editing) were examined.*
